# Supplementary material for: Natural proteome diversity links aneuploidy tolerance to protein turnover
Source: Nature. 2024 May 22;630(8015):149–57. doi: 10.1038/s41586-024-07442-9 (PMC11153158; doi:10.1038/s41586-024-07442-9)
Supplement: Supplementary file 1 — Supplementary Information guide containing descriptions for Supplementary Tables 1–20 and Supplementary Figs 1–2. [file 41586_2024_7442_MOESM1_ESM.pdf]

nature portfolio

https://doi.org/10.1038/s41586-024-07442-9

# Supplementary information

# Natural proteome diversity links aneuploidy tolerance to protein turnover

In the format provided by the authors and unedited

# Supplementary Information Guide

## List of SI Tables (provided separately)

SI Table 1: Strain annotations of natural isolates.

SI Table 2: Growth of natural isolates during cultivation for proteomics.

SI Table 3: Proteomes of 796 yeast isolates (non-imputed).

SI Table 4: Proteomes of disomic strains (non-imputed).

SI Table 5: List of strains showing a discrepancy between aneuploidy annotation as shown in SI Table 1 and the median gene copy number per chromosome as published in 10.1038/s41586-018-0030-5.

SI Table 6: List of strains showing a discrepancy between their median normalized chromosomal log2 mRNA ratios and the predicted median based on the aneuploidy annotation.

SI Table 7: Strains included in the integrated dataset.

SI Table 8: Gene-by-gene linear regression analysis of mRNA and protein attenuation in natural aneuploid isolates.

SI Table 9: Gene-by-gene linear regression analysis of mRNA and protein attenuation in lab-engineered synthetic disomic strains.

SI Table 10: Median mRNA or protein level attenuation of genes associated with KEGG cellular pathway terms, as well as GO biological process, GO molecular function, or GO cellular compartment terms.

SI Table 11: Quantification of dosage compensation across isolates.

SI Table 12: Strain-by-strain quantification of dosage compensation.

SI Table 13: Proteomes of two natural isolates (AMC, BBV) across different growth stages.

SI Table 14: Trans expression of genes in aneuploid natural isolates.

SI Table 15: Ubiquitinomics data of euploid and aneuploid natural isolates.

SI Table 16: Doubling times of natural isolates as measured during turnover experiments.

SI Table 17: Protein turnover data for 55 natural euploid and aneuploid isolates.

SI Table 18: Overview of the 1,046 samples included in the RNA-sequencing survey.

SI Table 19: Non-linear gradient ramp on nanoAcquity UPLC.

SI Table 20: Lower and upper m/z limits of the SWATH windows.

**Supplementary Fig. 1,** pages 1-20: Depiction of relative mRNA and protein levels as in Fig. 3a for each aneuploid natural isolate.

AAT, 1n, aneu;+1*1; mRNA

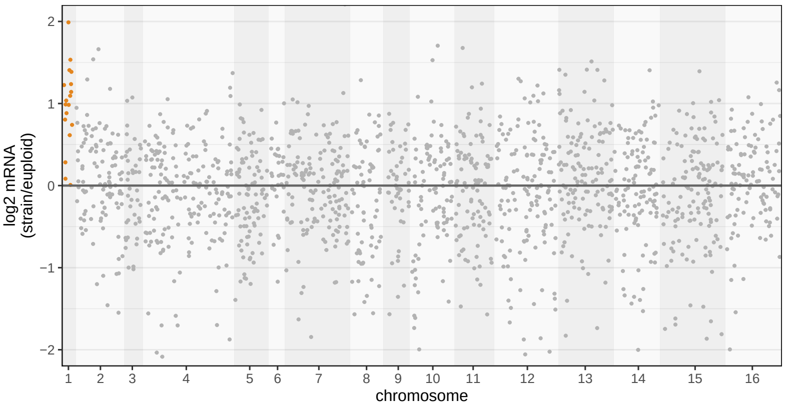


AAT, 1n, aneu;+1*1; protein

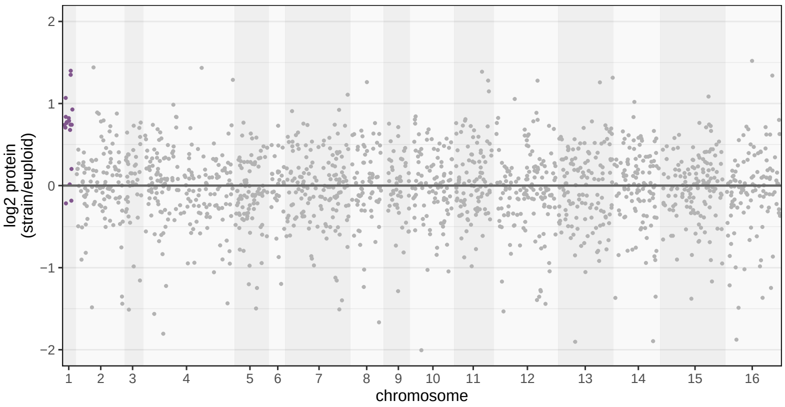


ABV, 3n, aneu;+1*1;+1*9; mRNA

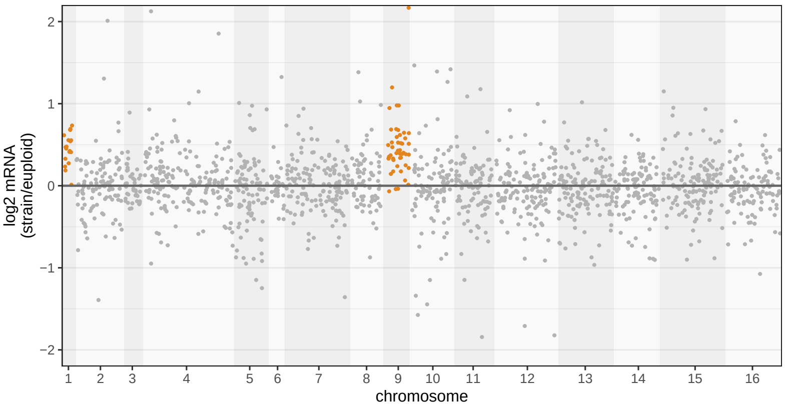


ABV, 3n, aneu;+1*1;+1*9; protein

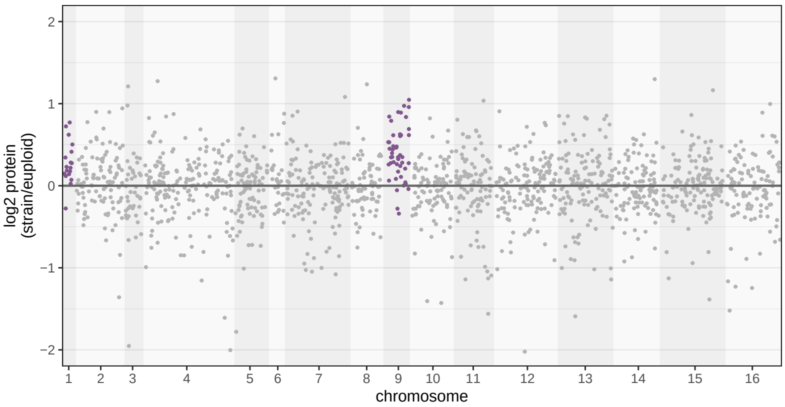


ACT, 1n, aneu;+1*1; mRNA

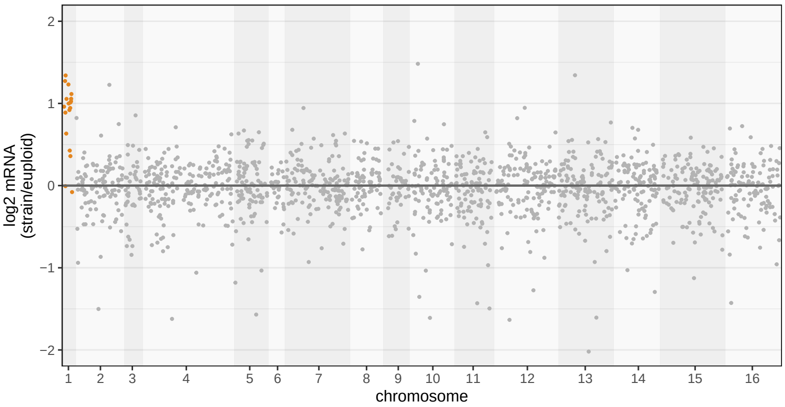


ACT, 1n, aneu;+1*1; protein

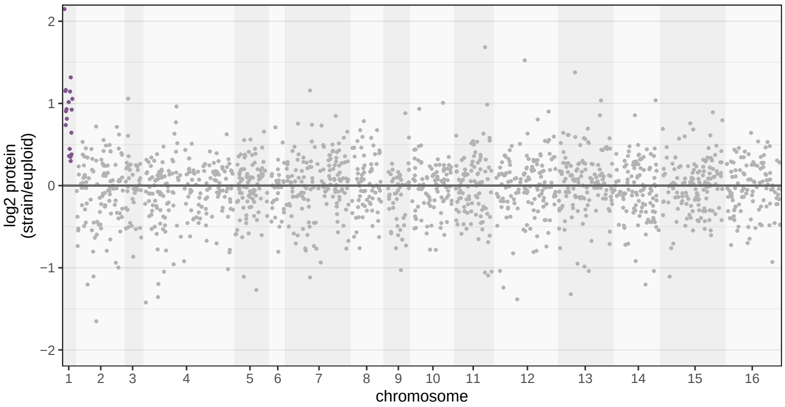


ACV, 1n, aneu;+1*1; mRNA

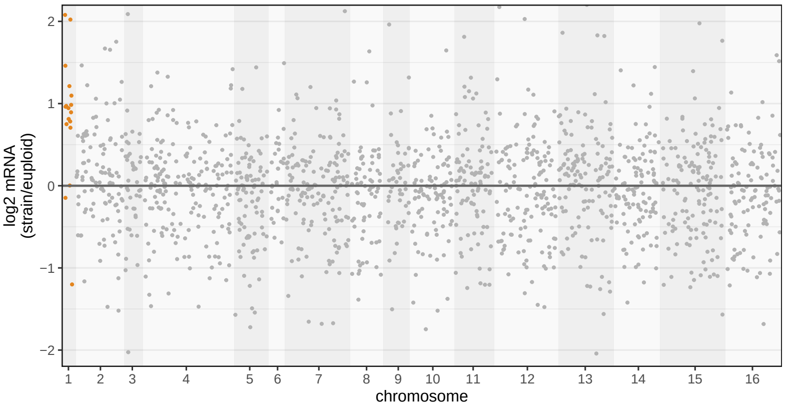


ACV, 1n, aneu;+1*1; protein

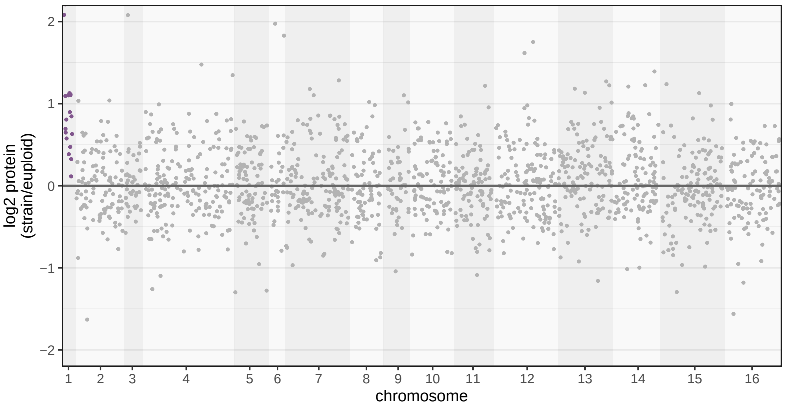


ADL, 2n, aneu;+1*1;+1*6;+1*9;+1*11; mRNA

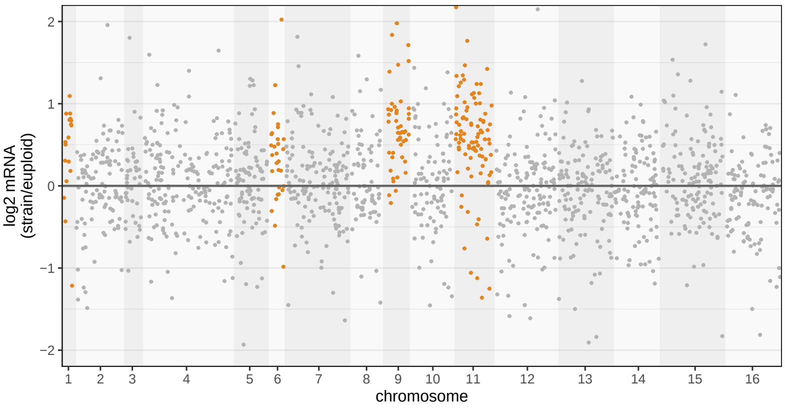


ADL, 2n, aneu;+1*1;+1*6;+1*9;+1*11; protein

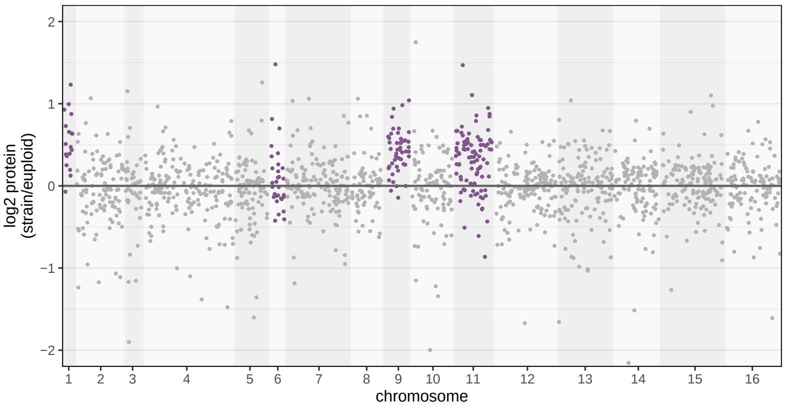

AEE, 3n, aneu;+2*1;+1*5;+1*8;+1*9; mRNA

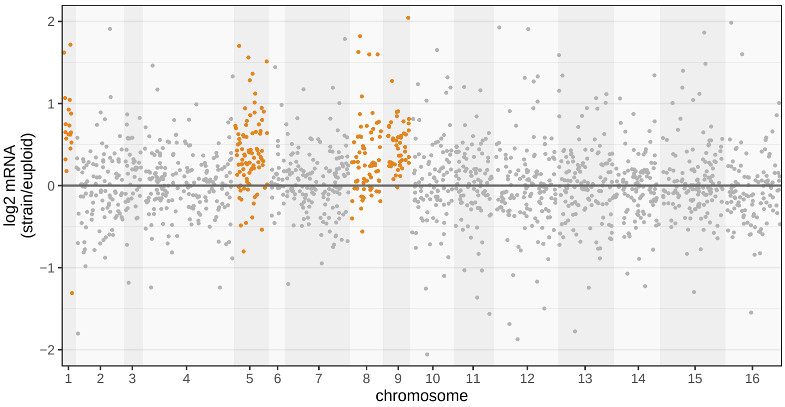


AEE, 3n, aneu;+2*1;+1*5;+1*8;+1*9; protein

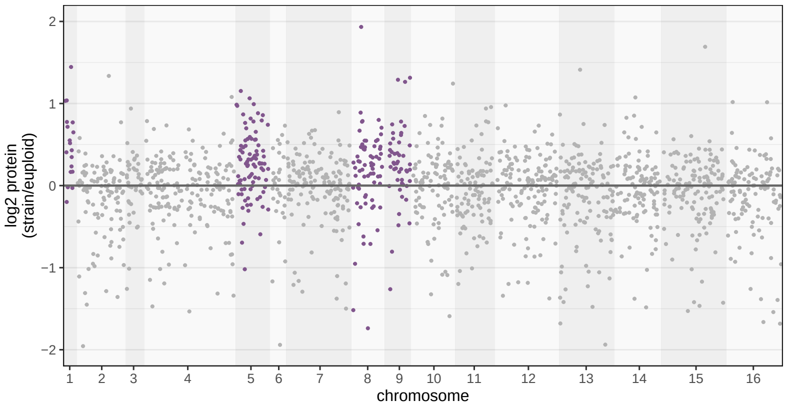


AFR, 1n, aneu;+1*1; mRNA

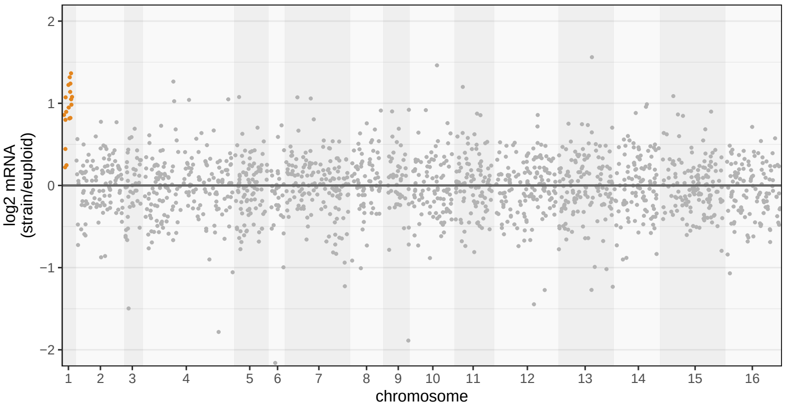


AFR, 1n, aneu;+1*1; protein

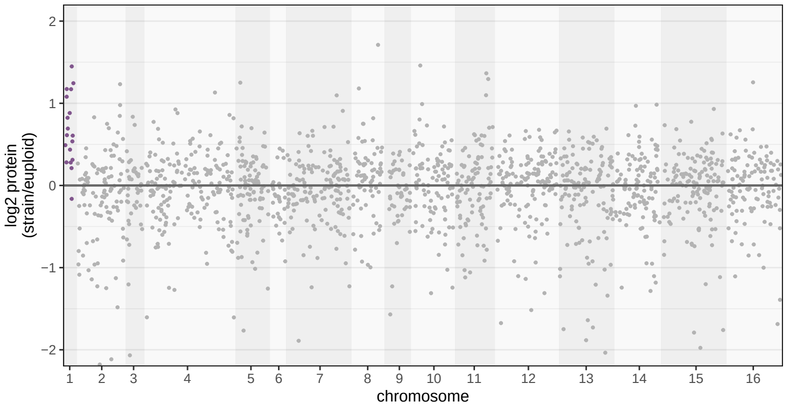


AGP, 1n, aneu;+1*1;+1*9; mRNA

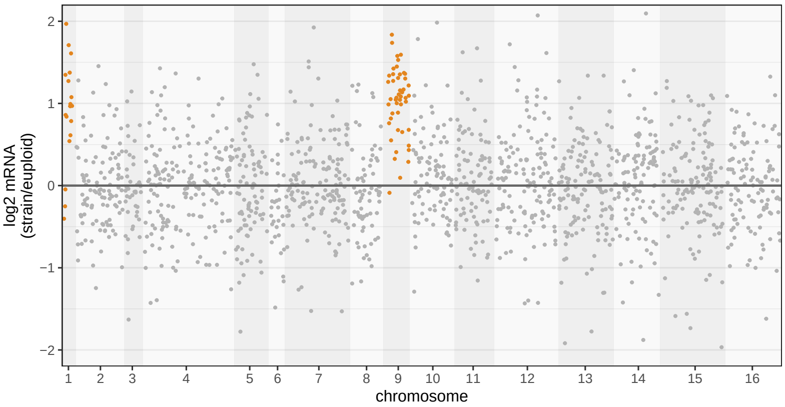


AGP, 1n, aneu;+1*1;+1*9; protein

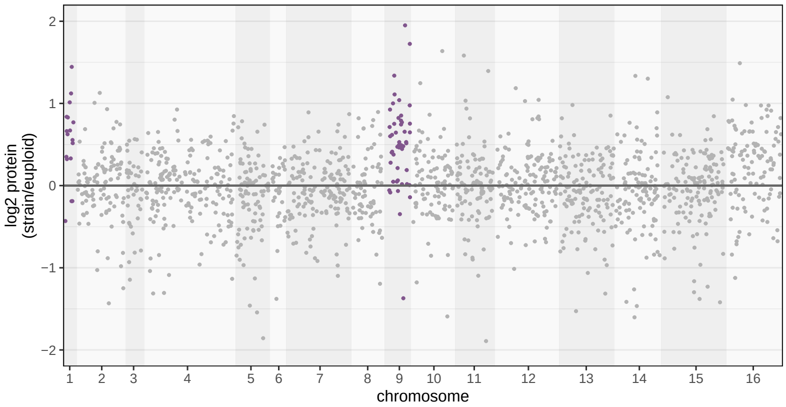


AHD, 1n, aneu;+1*1; mRNA

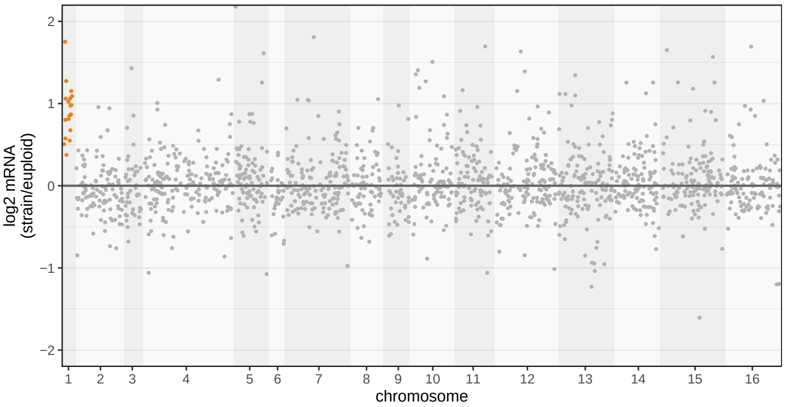


AHD, 1n, aneu;+1*1; protein

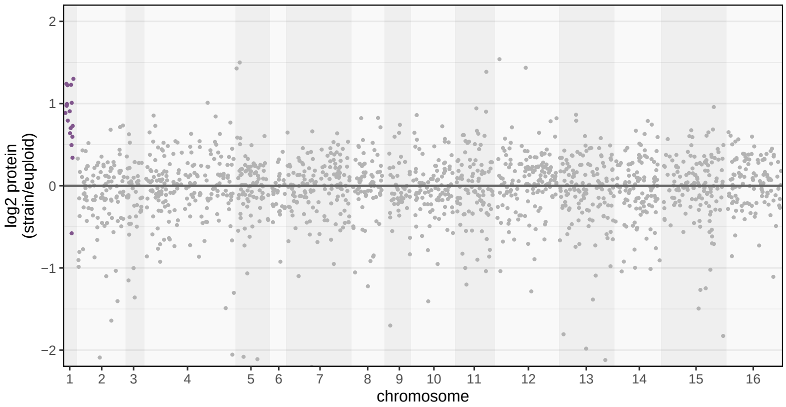


AHS, 1n, aneu;+1*9; mRNA

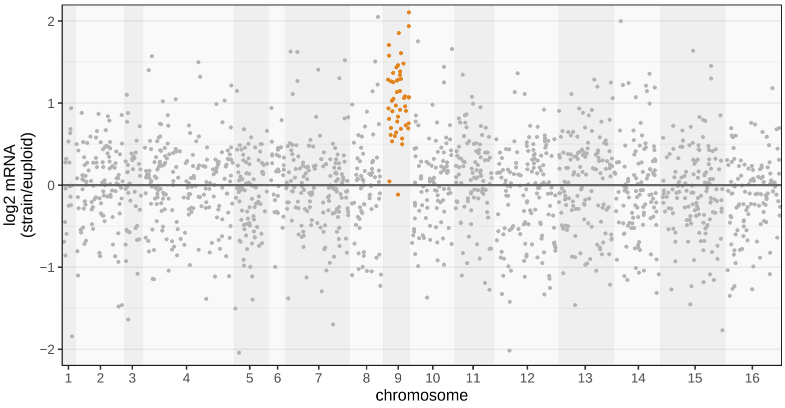


AHS, 1n, aneu;+1*9; protein

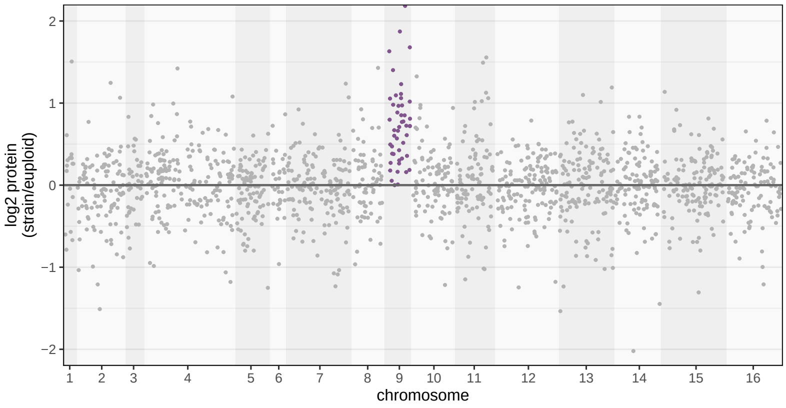

AIE, 1n, aneu;+1*9; mRNA

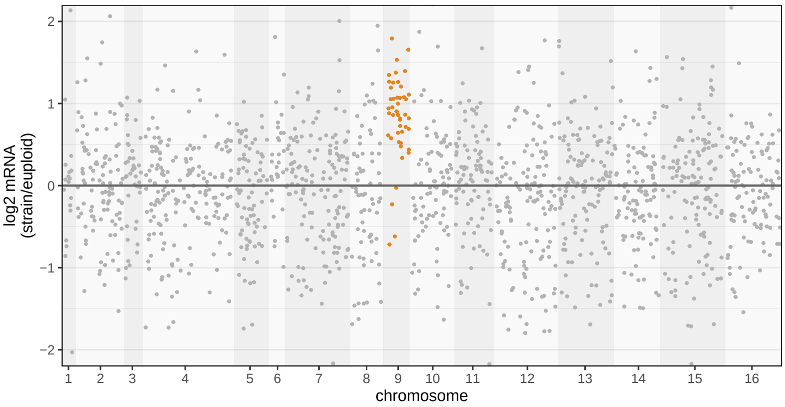


AIE, 1n, aneu;+1*9; protein

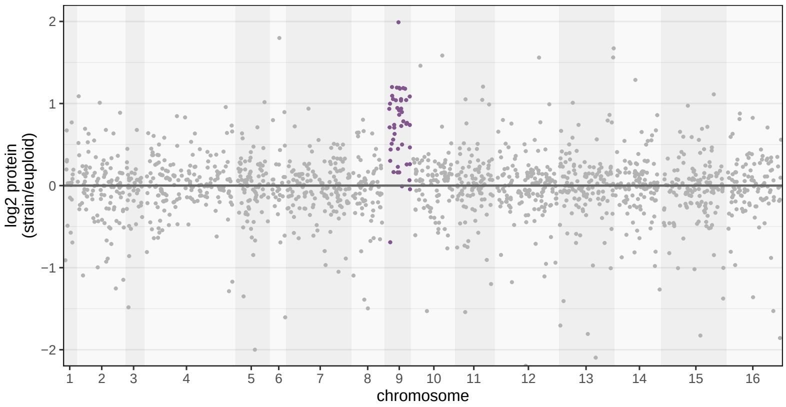


AII, 2n, aneu;+1*1; mRNA

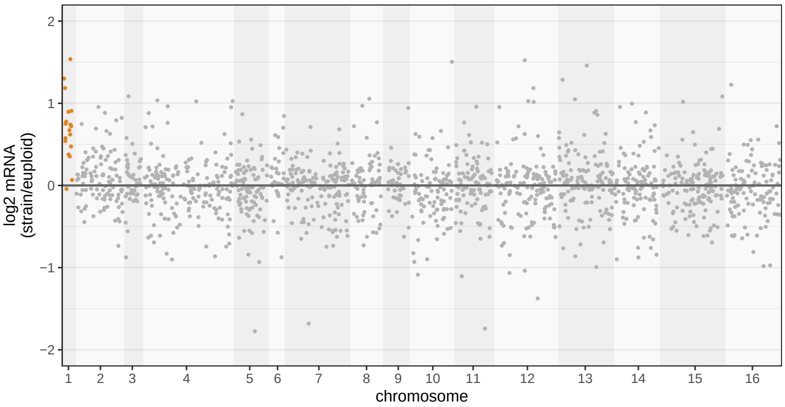


AII, 2n, aneu;+1*1; protein

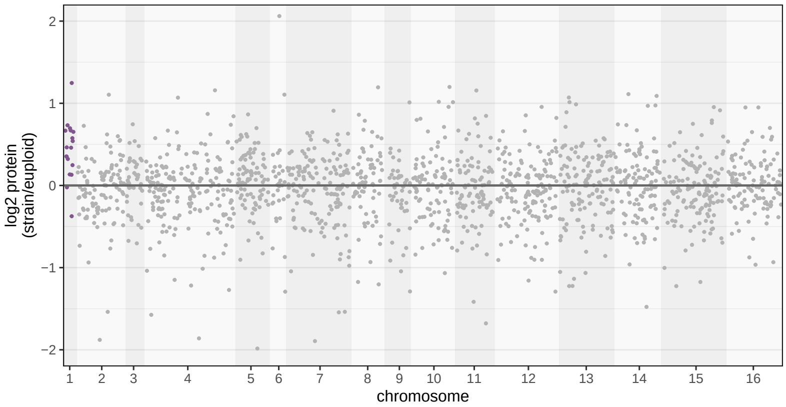


AIP, 2n, aneu;+1*9; mRNA

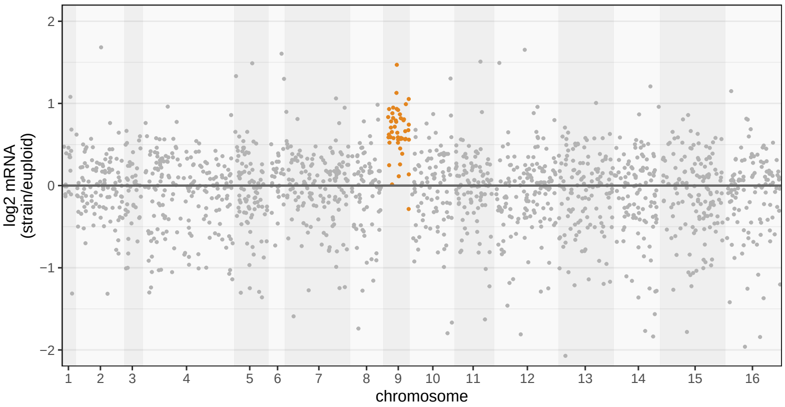


AIP, 2n, aneu;+1*9; protein

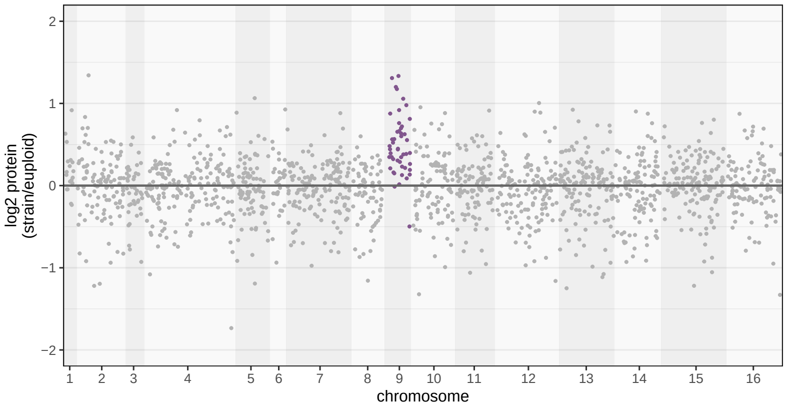


ALK, 2n, aneu;+1*5; mRNA

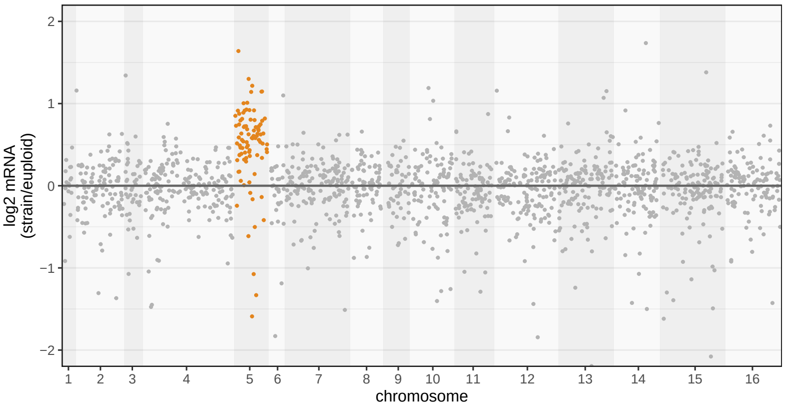


ALK, 2n, aneu;+1*5; protein

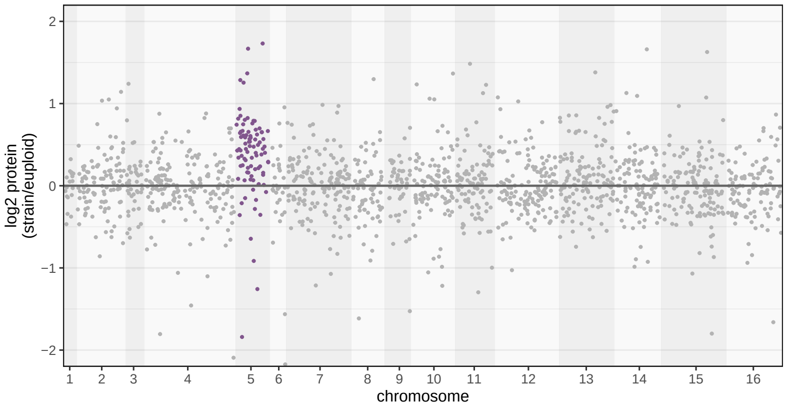


ALM, 2n, aneu;+1*9; mRNA

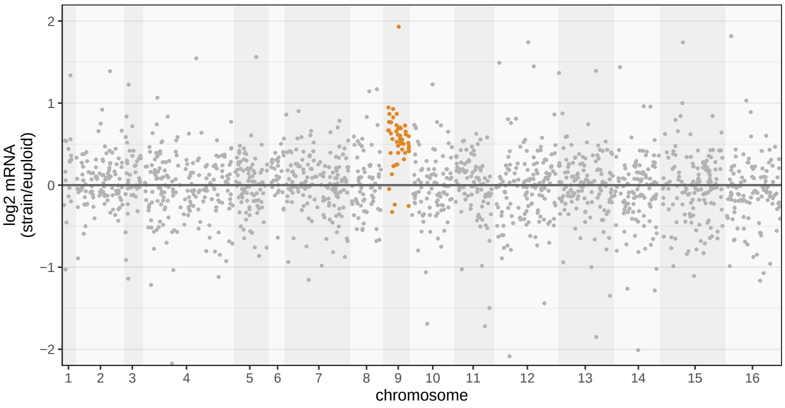


ALM, 2n, aneu;+1*9; protein

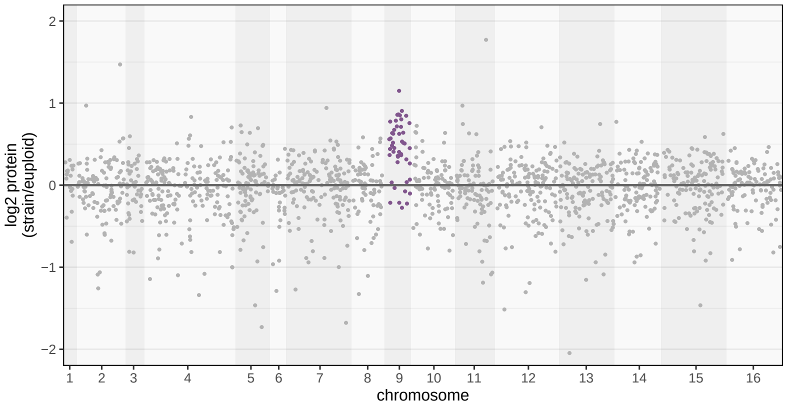

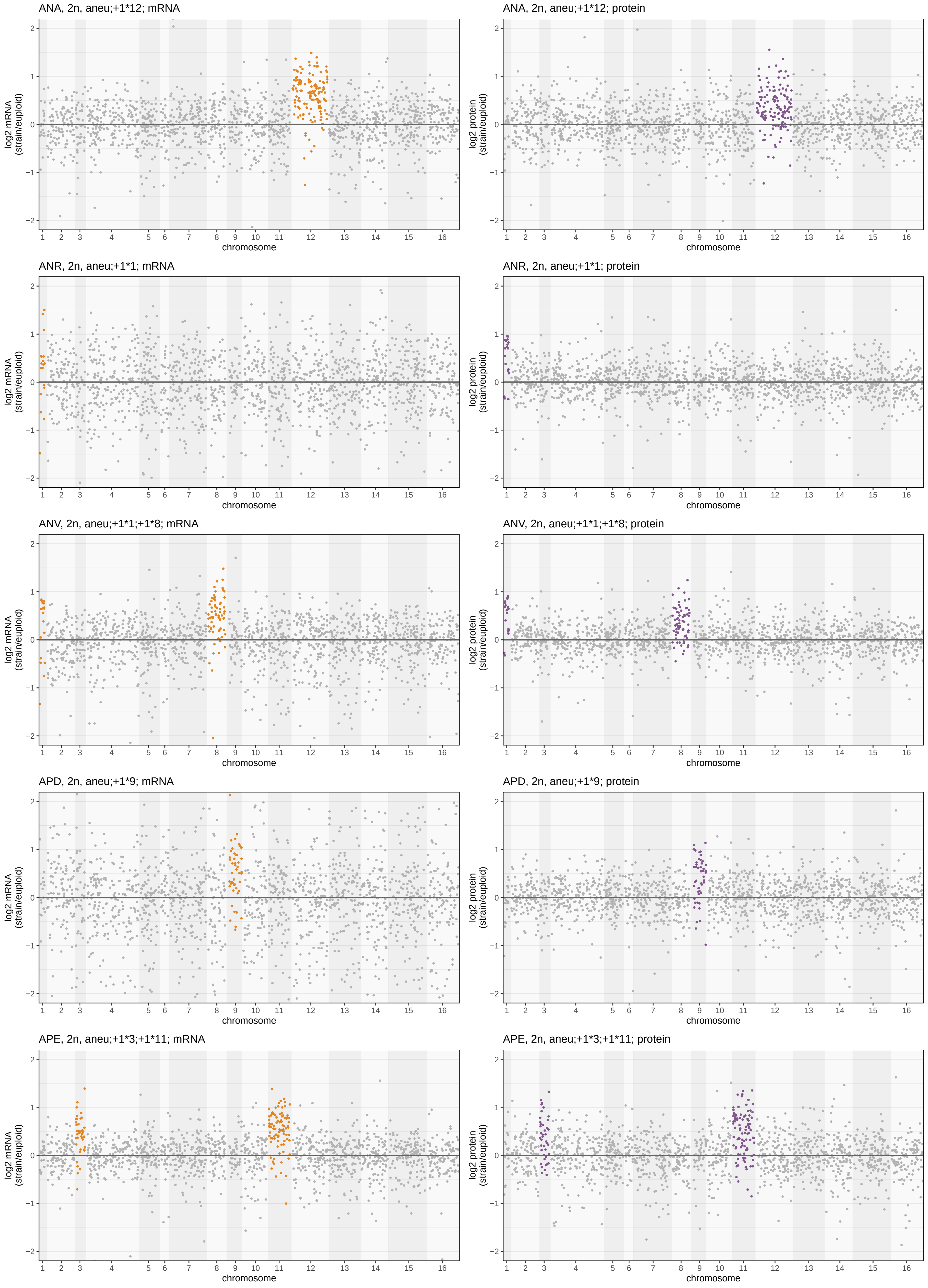

ANA, 2n, aneu;+1*12; mRNA
ANA, 2n, aneu;+1*12; protein
ANR, 2n, aneu;+1*1; mRNA
ANR, 2n, aneu;+1*1; protein
ANV, 2n, aneu;+1*1;+1*8; mRNA
ANV, 2n, aneu;+1*1;+1*8; protein
APD, 2n, aneu;+1*9; mRNA
APD, 2n, aneu;+1*9; protein
APE, 2n, aneu;+1*3;+1*11; mRNA
APE, 2n, aneu;+1*3;+1*11; protein
log2 mRNA (strain/euploid)
log2 protein (strain/euploid)
chromosome
1 2 3 4 5 6 7 8 9 10 11 12 13 14 15 16
−2 −1 0 1 2

API, 2n, aneu;+1*8;+1*9; mRNA

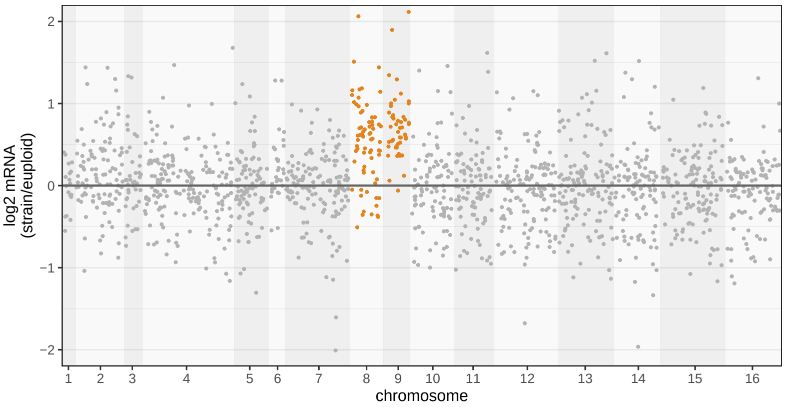


API, 2n, aneu;+1*8;+1*9; protein

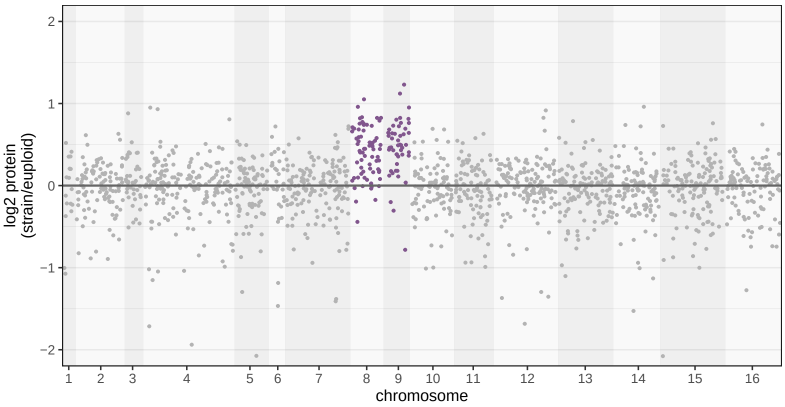


APM, 2n, aneu;+1*9; mRNA

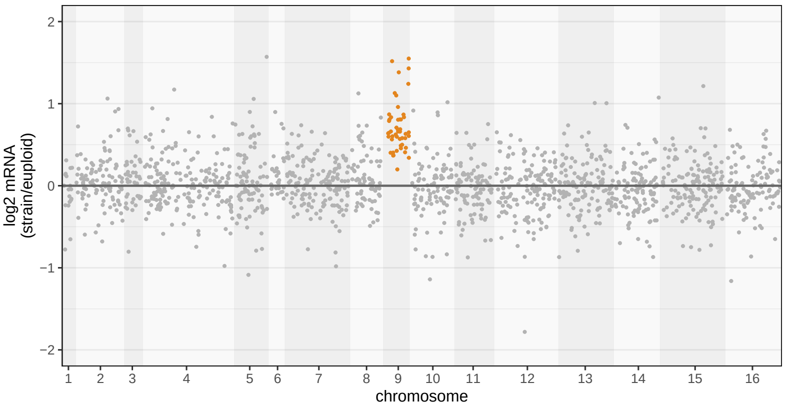


APM, 2n, aneu;+1*9; protein

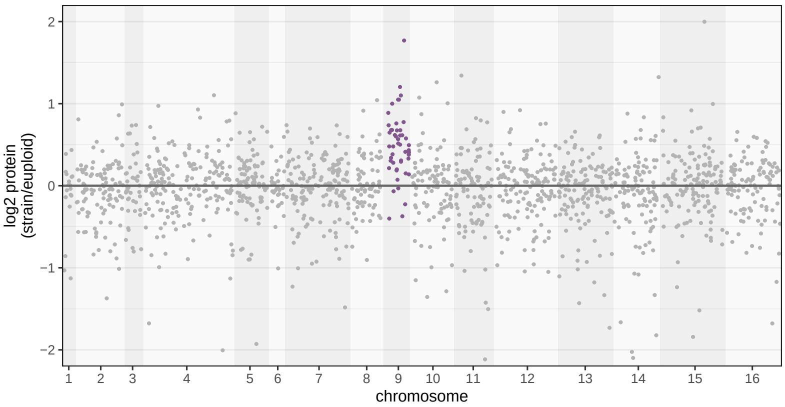


APN, 3n, aneu;+1*1; mRNA

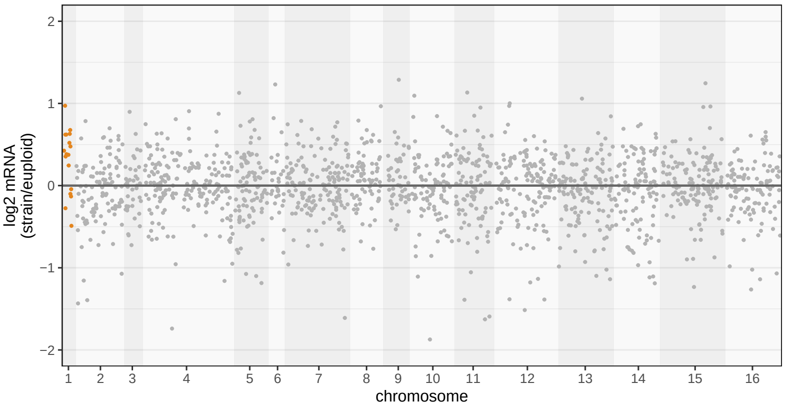


APN, 3n, aneu;+1*1; protein

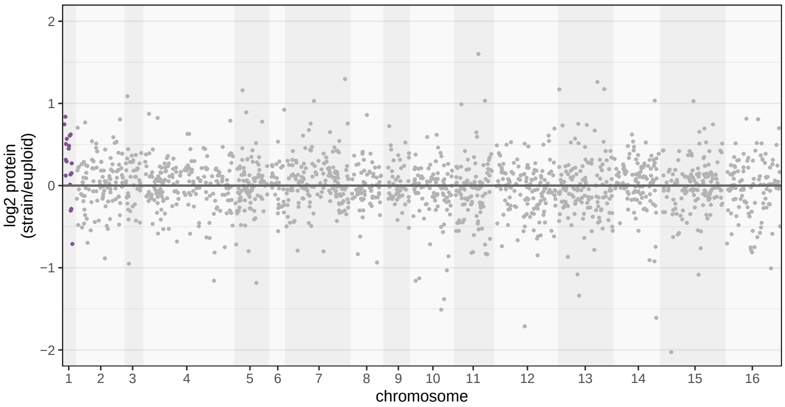


APT, 2n, aneu;+1*14; mRNA

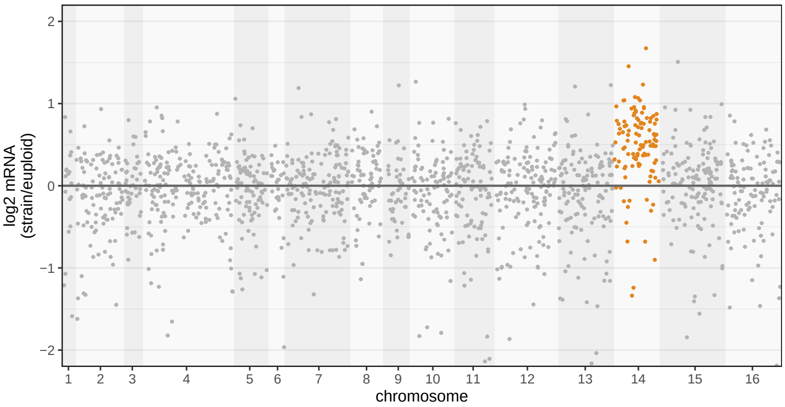


APT, 2n, aneu;+1*14; protein

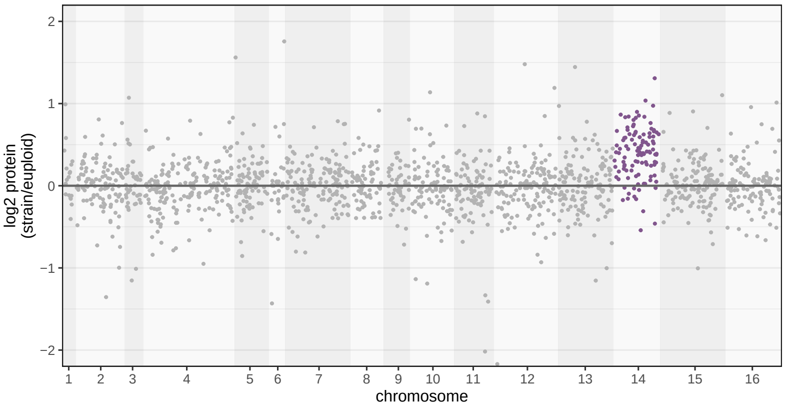


AQD, 2n, aneu;+1*8; mRNA

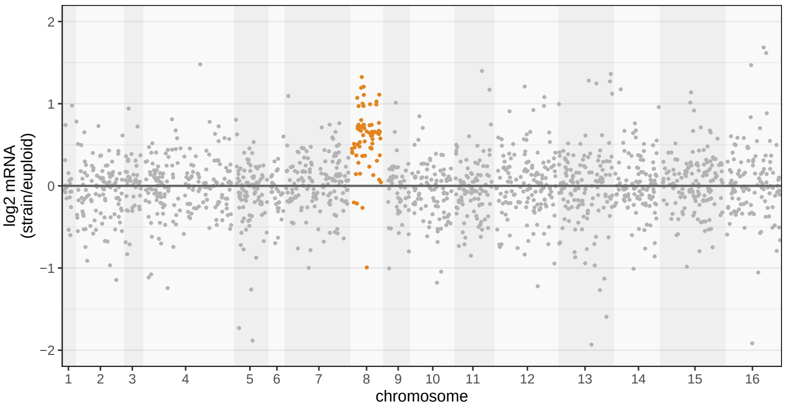


AQD, 2n, aneu;+1*8; protein

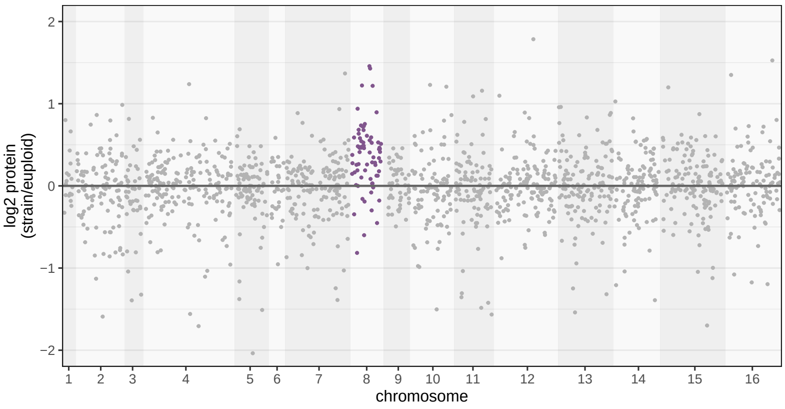

AQM, 2n, aneu;+1*9;+1*16; mRNA

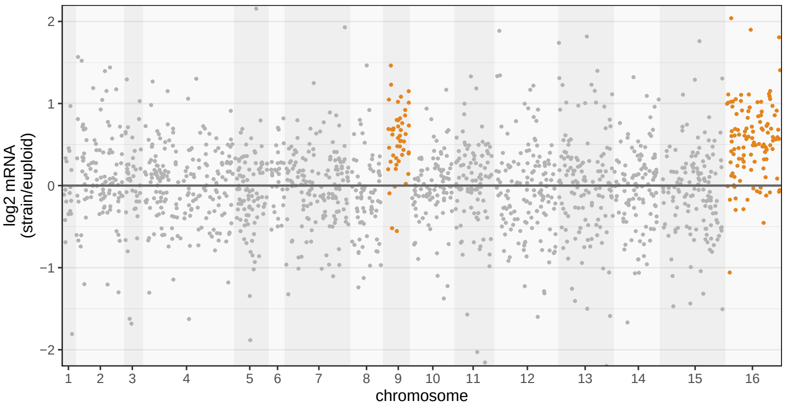


AQM, 2n, aneu;+1*9;+1*16; protein

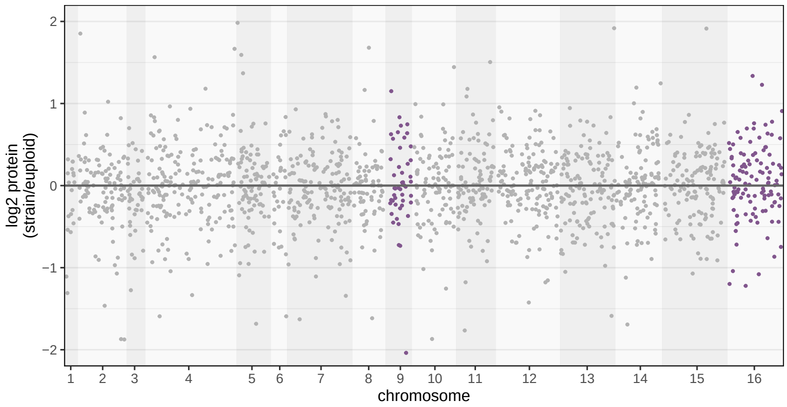


AQQ, 2n, aneu;+1*9;+1*14; mRNA

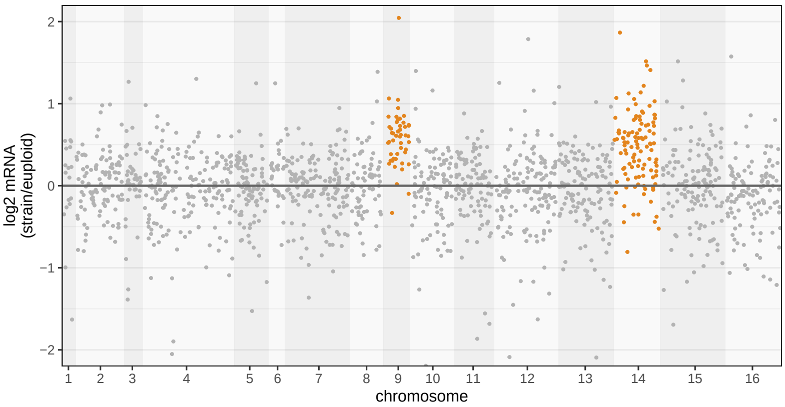


AQQ, 2n, aneu;+1*9;+1*14; protein

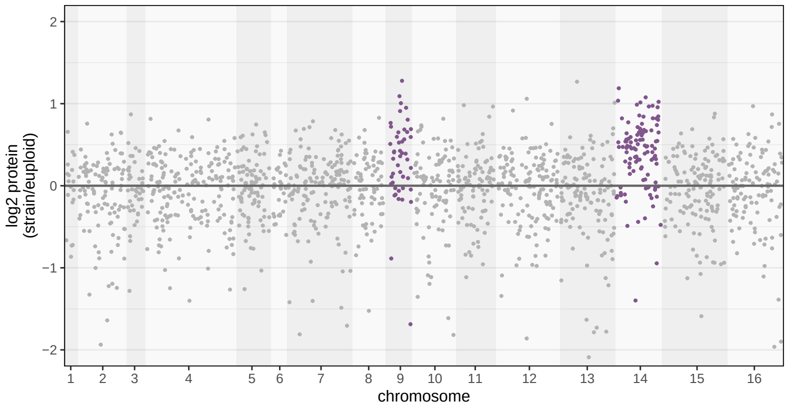


AQR, 2n, aneu;+1*11;+1*14; mRNA

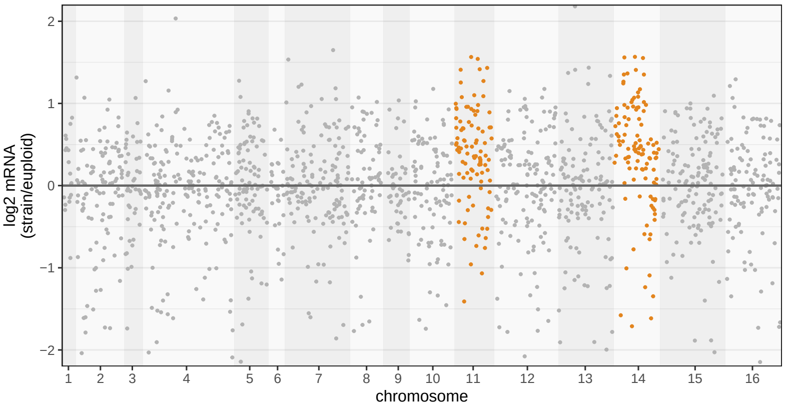


AQR, 2n, aneu;+1*11;+1*14; protein

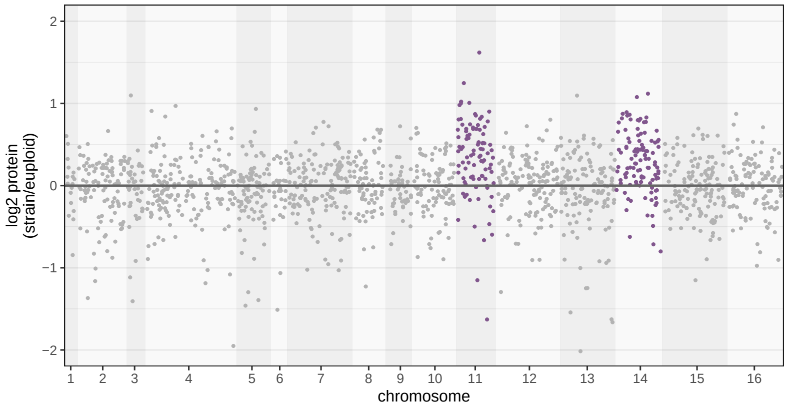


ARL, 2n, aneu;+1*1; mRNA

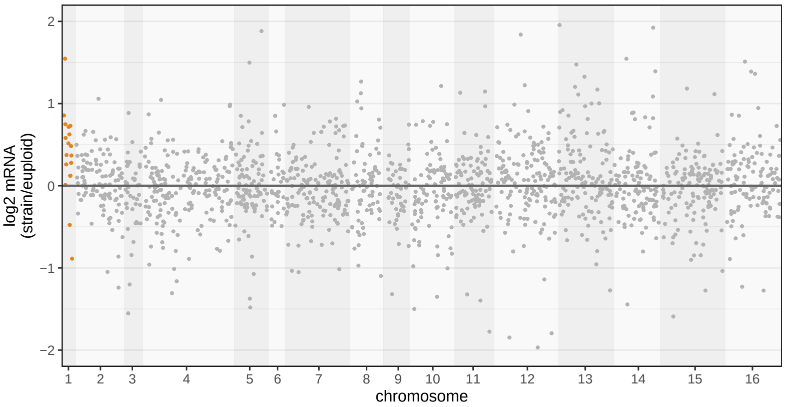


ARL, 2n, aneu;+1*1; protein

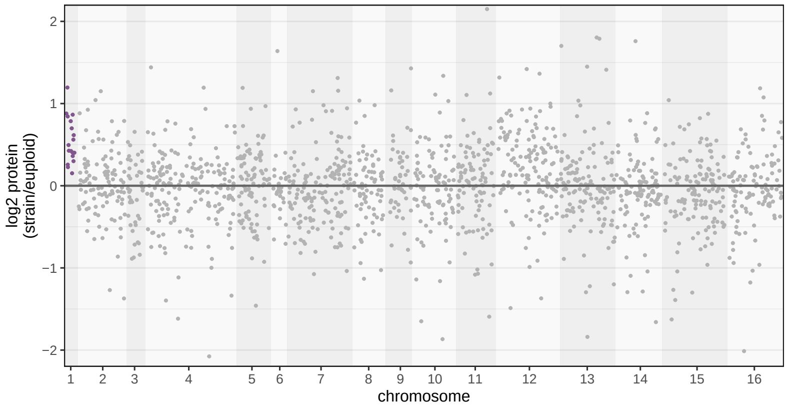


ARS, 2n, aneu;+1*1; mRNA

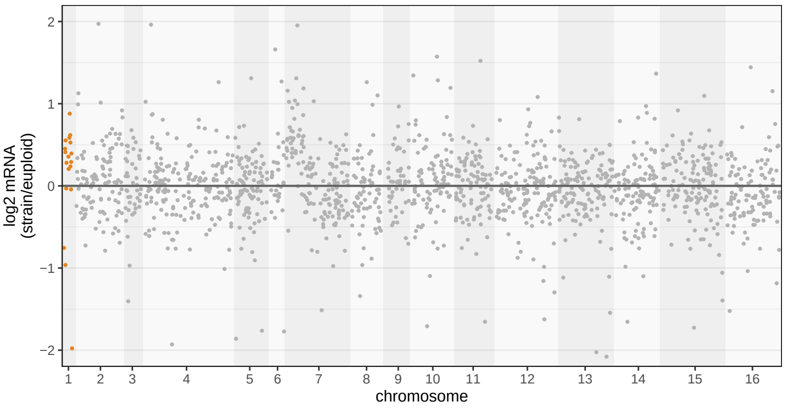


ARS, 2n, aneu;+1*1; protein

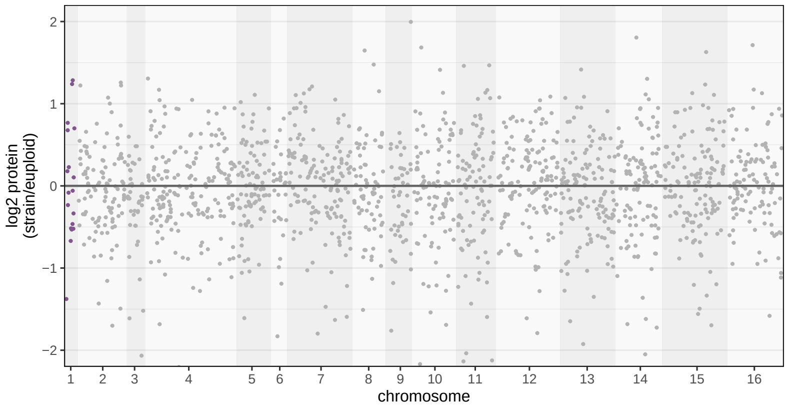

ARV, 2n, aneu;+1*1; mRNA

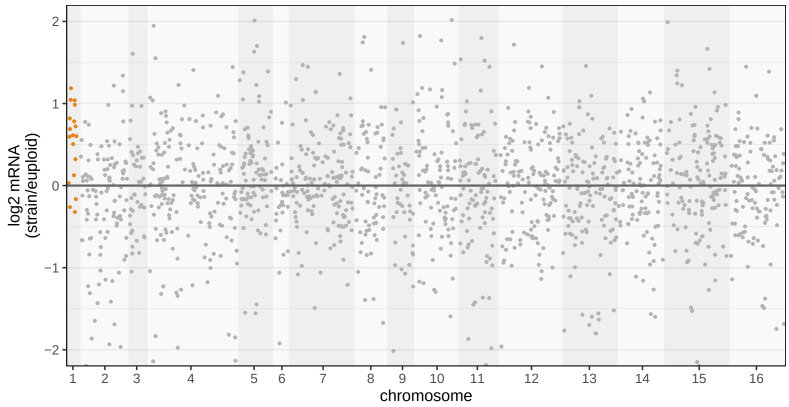


ARV, 2n, aneu;+1*1; protein

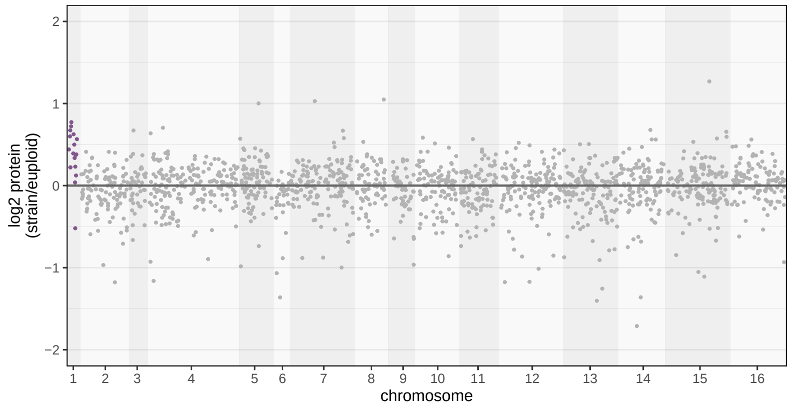


ASV, 2n, aneu;+1*9; mRNA

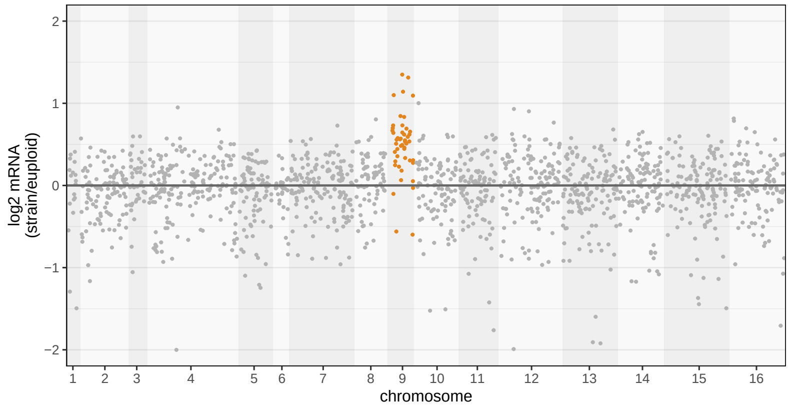


ASV, 2n, aneu;+1*9; protein

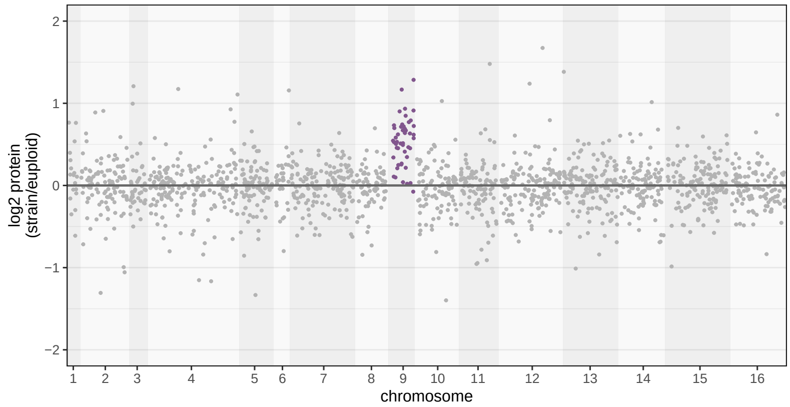


ATC, 2n, aneu;+1*11; mRNA

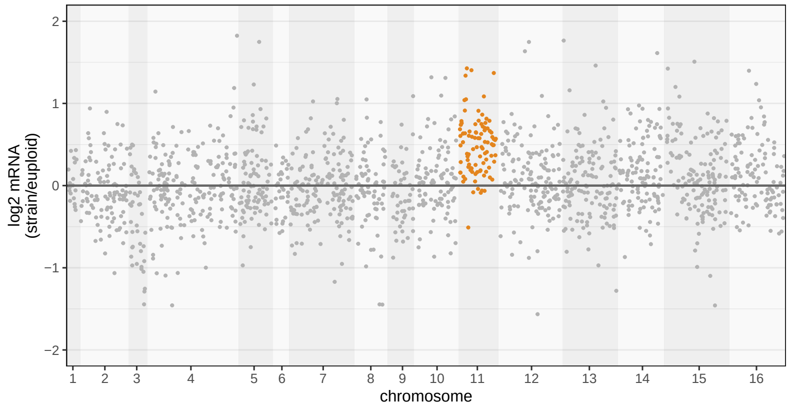


ATC, 2n, aneu;+1*11; protein

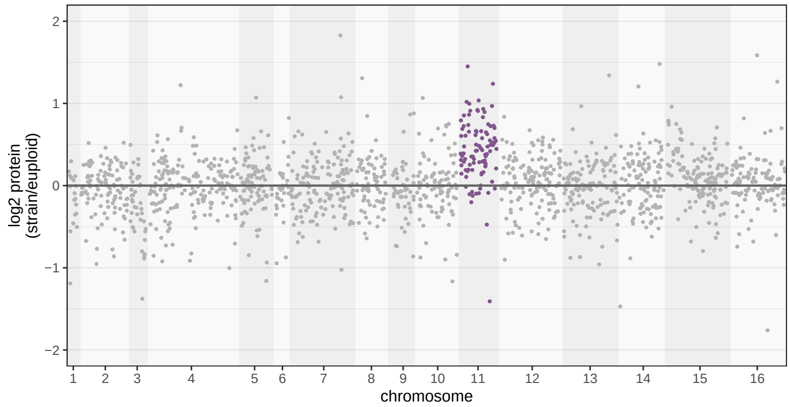


AVC, 2n, aneu;−1*1;+1*9;+1*15; mRNA

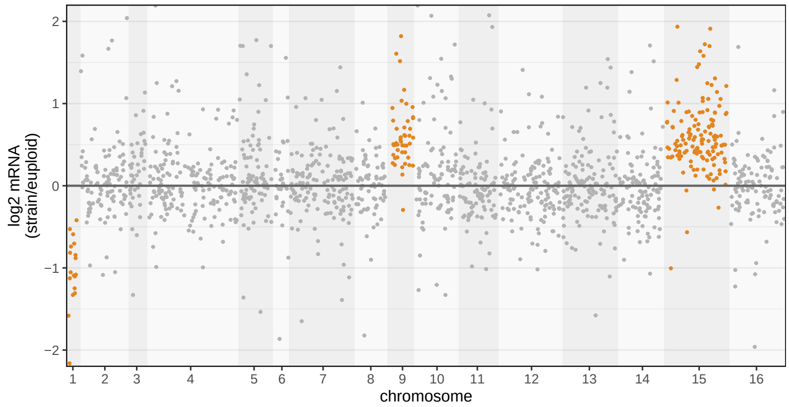


AVC, 2n, aneu;−1*1;+1*9;+1*15; protein

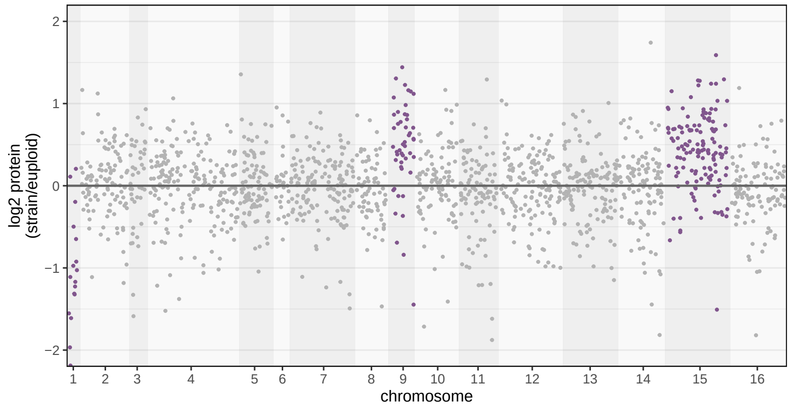


AVD, 2n, aneu;+1*3;+1*11; mRNA

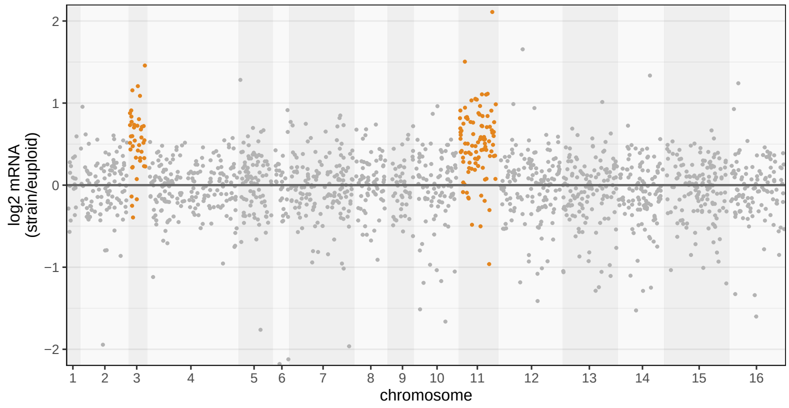


AVD, 2n, aneu;+1*3;+1*11; protein

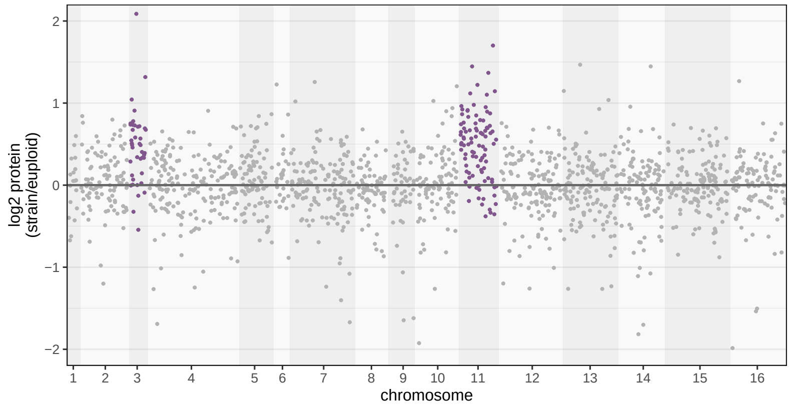

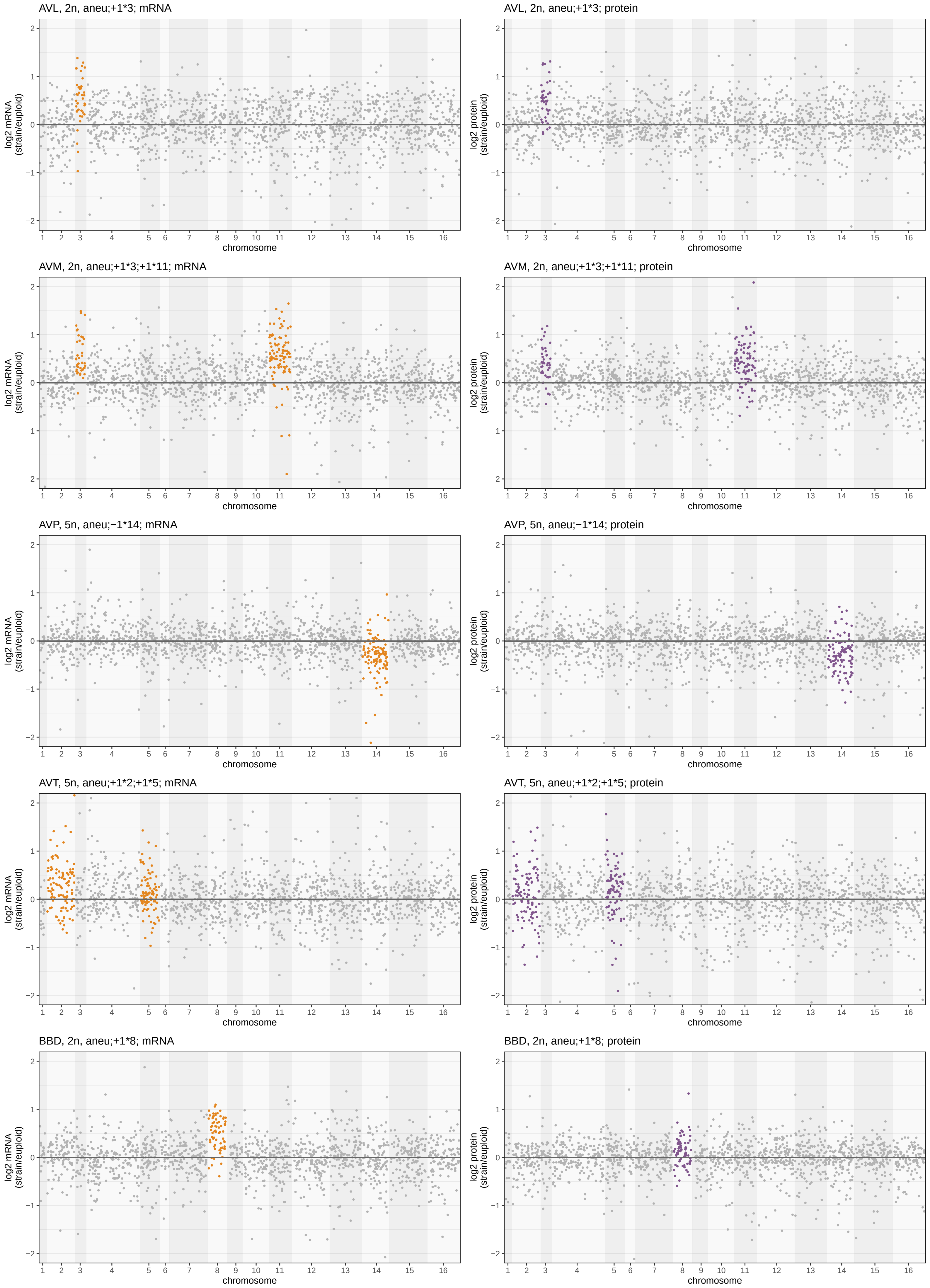
AVL, 2n, aneu;+1*3; mRNA
AVL, 2n, aneu;+1*3; protein
AVM, 2n, aneu;+1*3;+1*11; mRNA
AVM, 2n, aneu;+1*3;+1*11; protein
AVP, 5n, aneu;−1*14; mRNA
AVP, 5n, aneu;−1*14; protein
AVT, 5n, aneu;+1*2;+1*5; mRNA
AVT, 5n, aneu;+1*2;+1*5; protein
BBD, 2n, aneu;+1*8; mRNA
BBD, 2n, aneu;+1*8; protein
log2 mRNA (strain/euploid)
log2 protein (strain/euploid)
chromosome
1 2 3 4 5 6 7 8 9 10 11 12 13 14 15 16
−2 −1 0 1 2

BBV, 2n, aneu;+1*3; mRNA

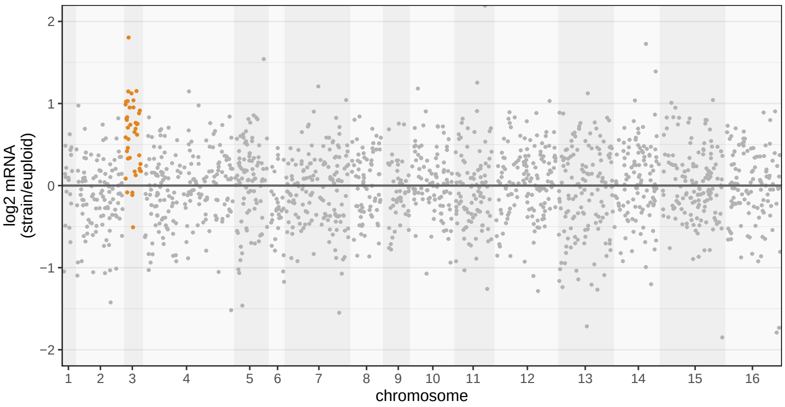


BBV, 2n, aneu;+1*3; protein

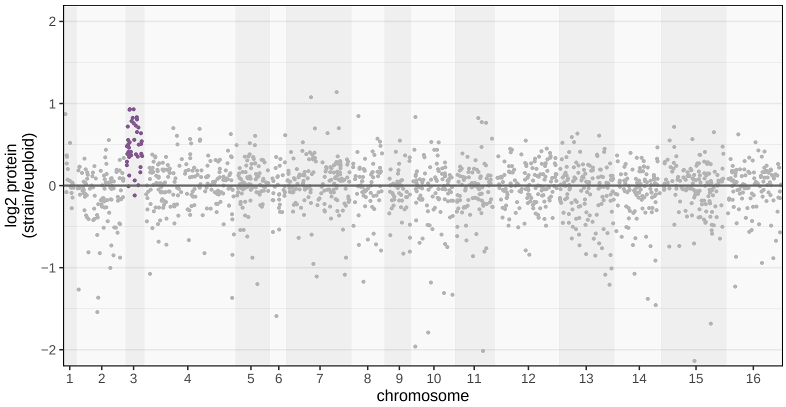


BDA, 2n, aneu;+1*1; mRNA

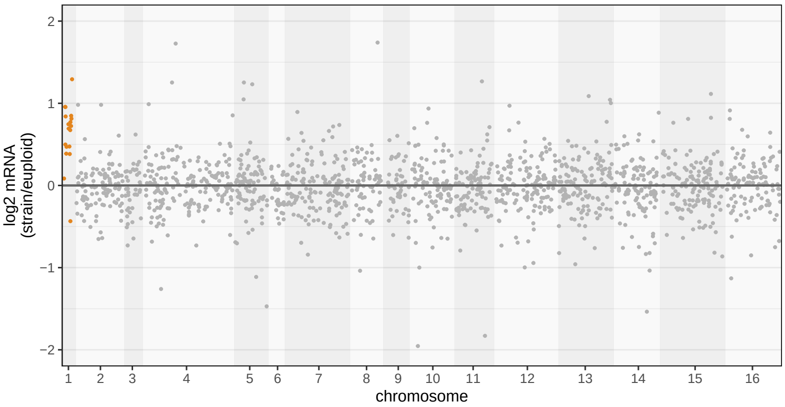


BDA, 2n, aneu;+1*1; protein

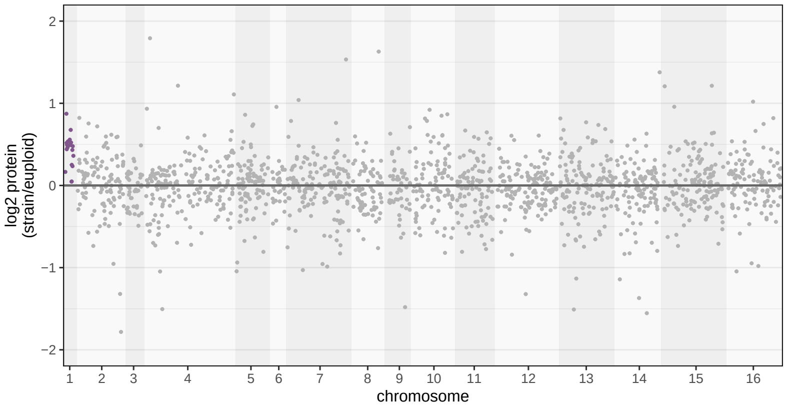


BDE, 3n, aneu;−1*3; mRNA

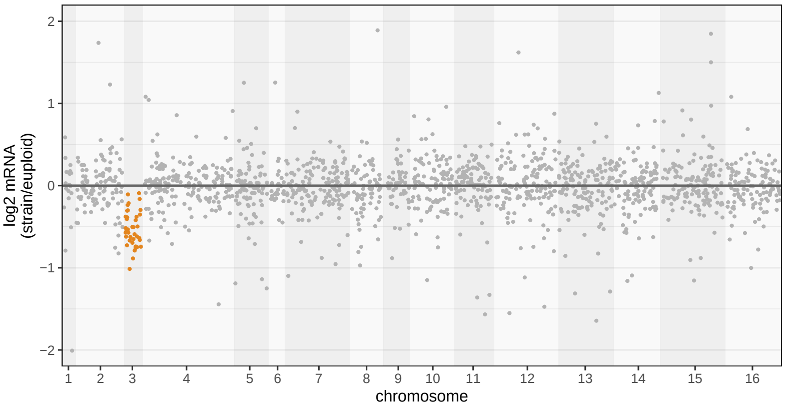


BDE, 3n, aneu;−1*3; protein

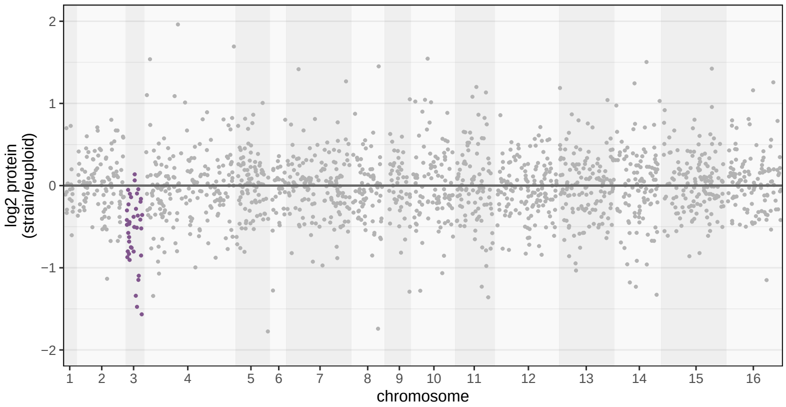


BDI, 2n, aneu;+1*1; mRNA

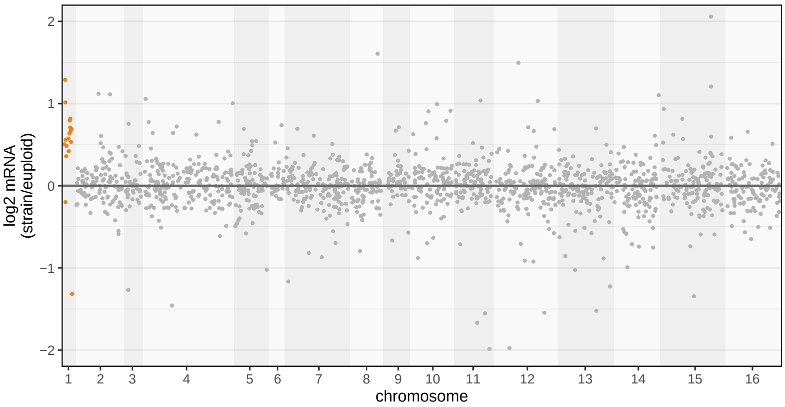


BDI, 2n, aneu;+1*1; protein

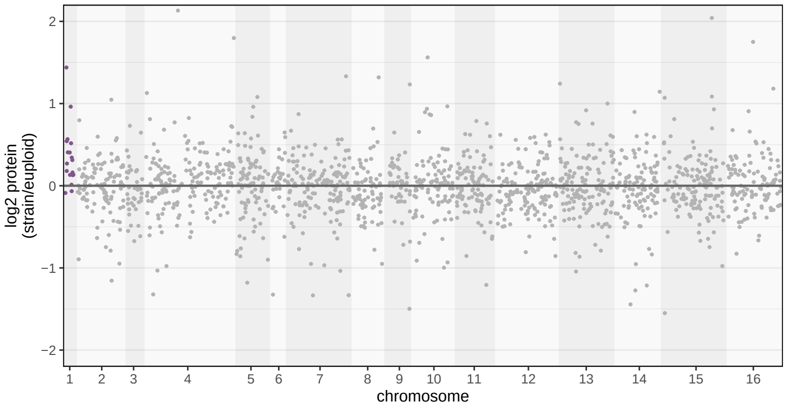


BDK, 2n, aneu;+1*1; mRNA

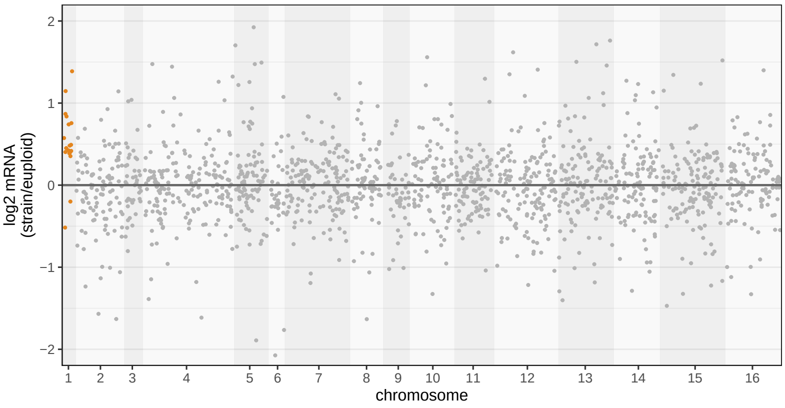


BDK, 2n, aneu;+1*1; protein

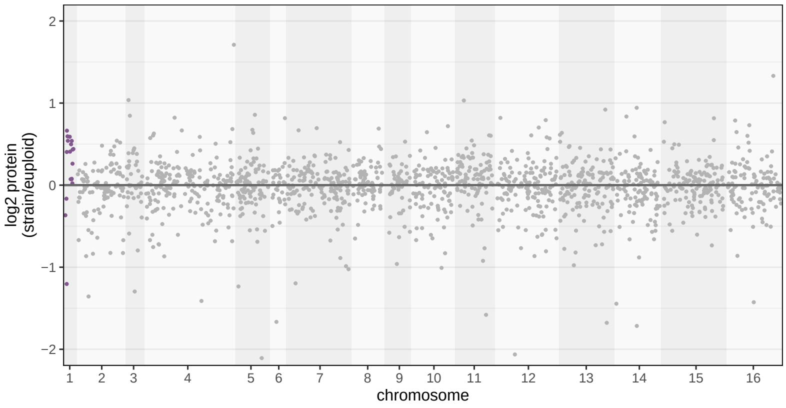

BEC, 2n, aneu;+1*12; mRNA

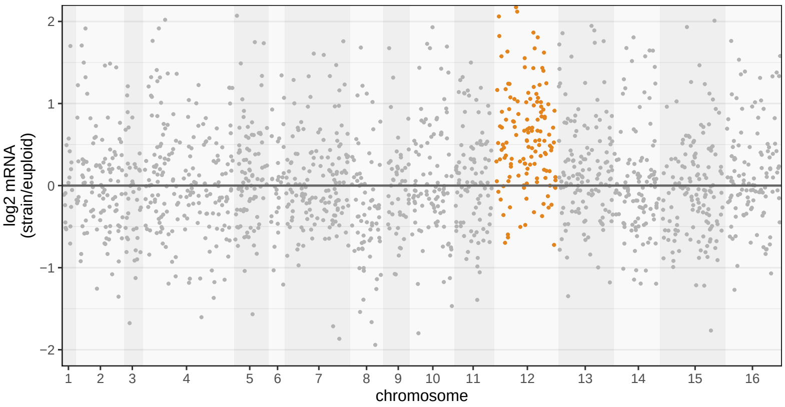


BEC, 2n, aneu;+1*12; protein

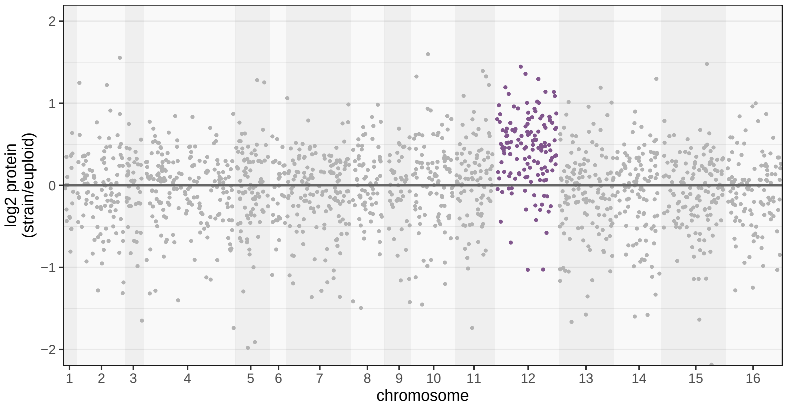


BFM, 4n, aneu;−1*13;−1*16; mRNA

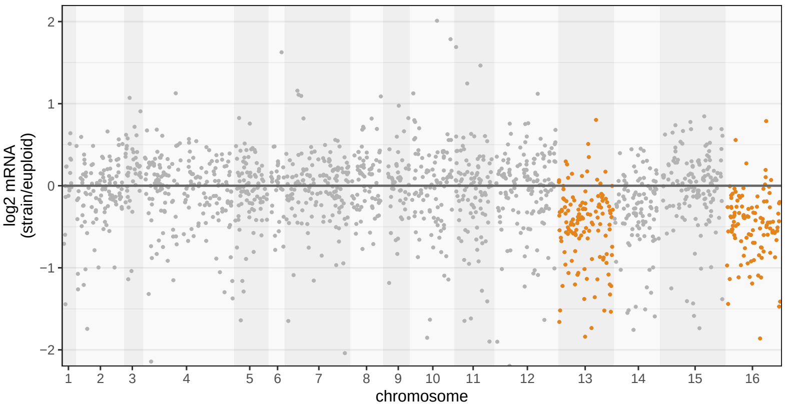


BFM, 4n, aneu;−1*13;−1*16; protein

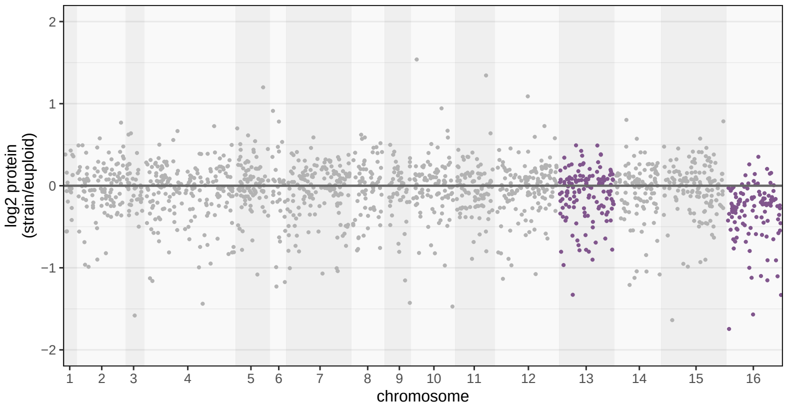


BFV, 2n, aneu;+1*11; mRNA

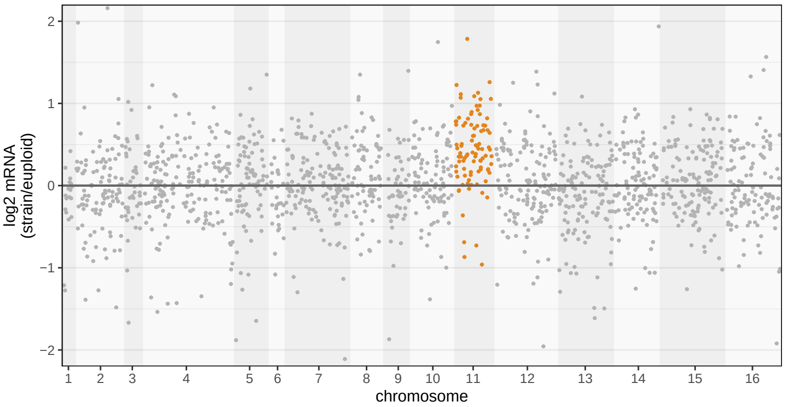


BFV, 2n, aneu;+1*11; protein

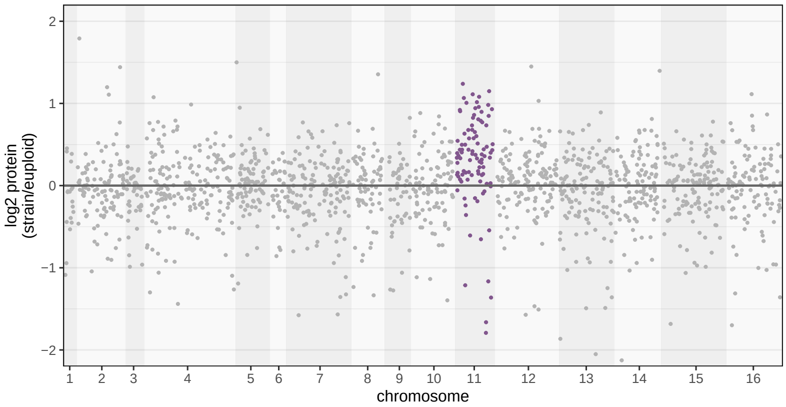


BGS, 2n, aneu;+1*10; mRNA

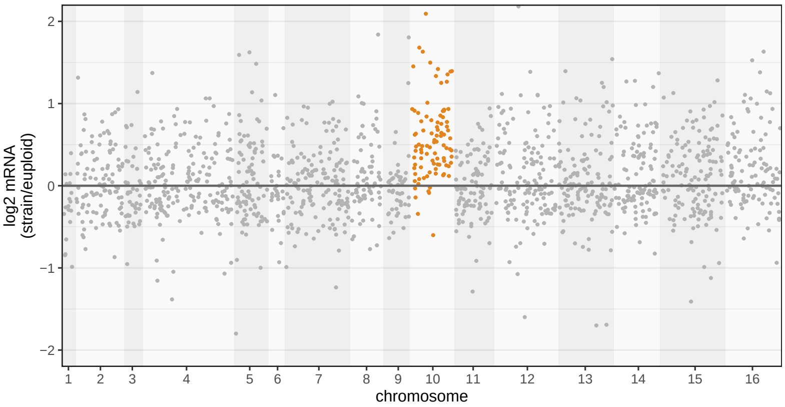


BGS, 2n, aneu;+1*10; protein

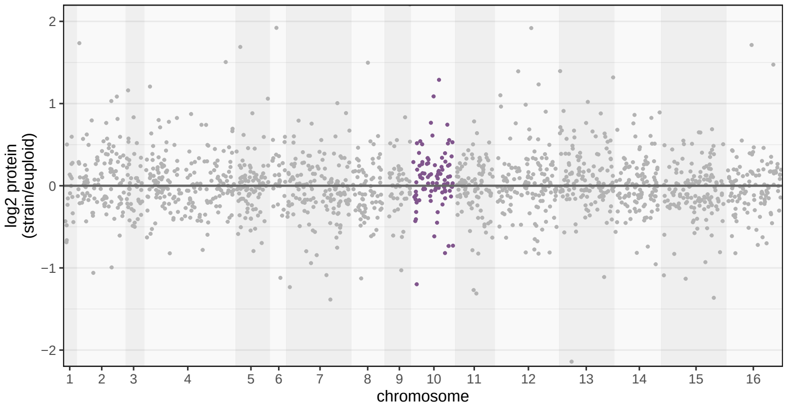


BHE, 2n, aneu;+1*14; mRNA

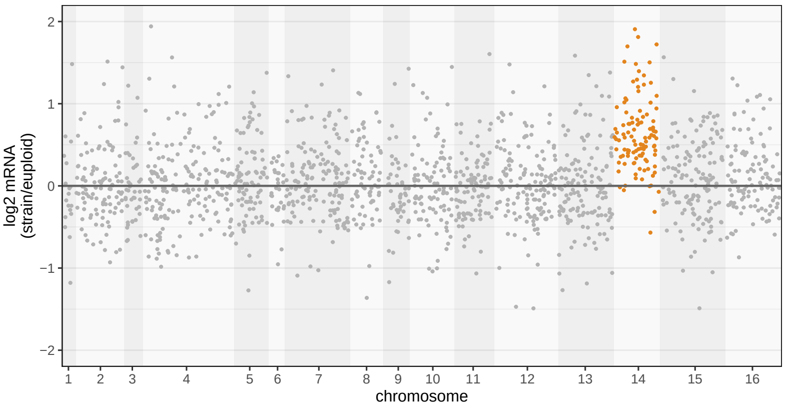


BHE, 2n, aneu;+1*14; protein

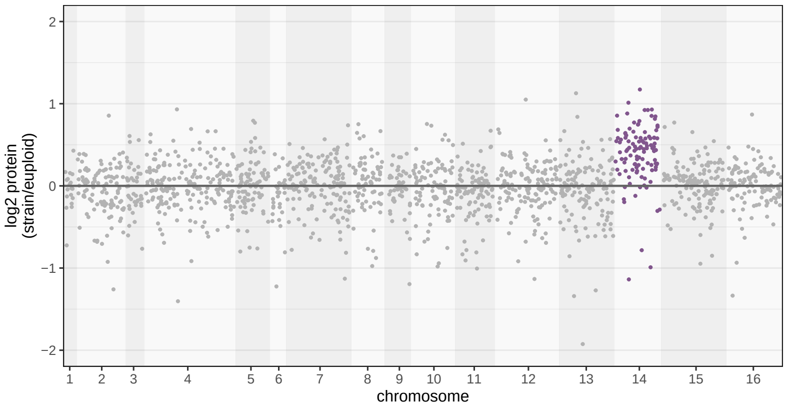

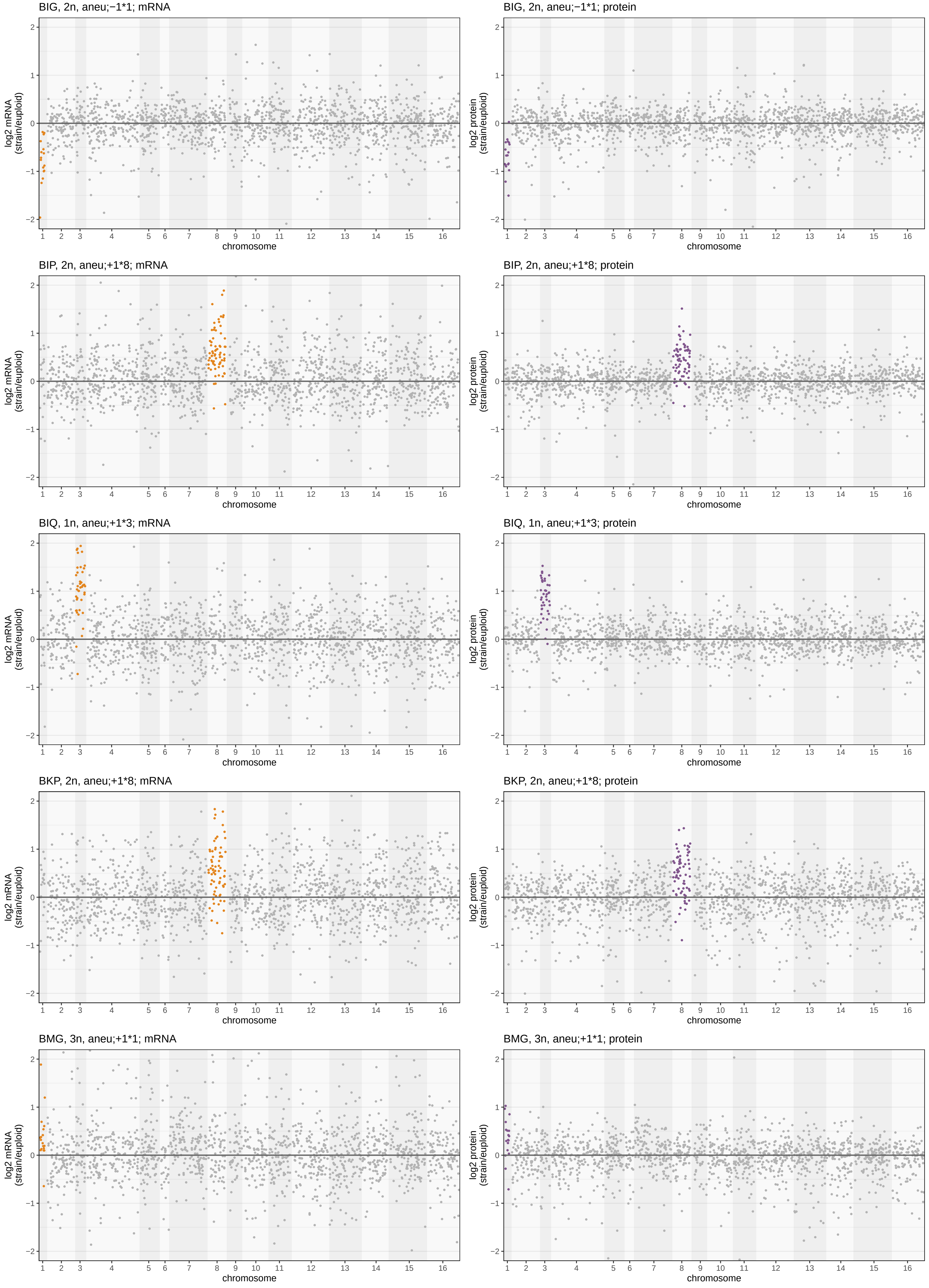

BIG, 2n, aneu;−1*1; mRNA
BIG, 2n, aneu;−1*1; protein
BIP, 2n, aneu;+1*8; mRNA
BIP, 2n, aneu;+1*8; protein
BIQ, 1n, aneu;+1*3; mRNA
BIQ, 1n, aneu;+1*3; protein
BKP, 2n, aneu;+1*8; mRNA
BKP, 2n, aneu;+1*8; protein
BMG, 3n, aneu;+1*1; mRNA
BMG, 3n, aneu;+1*1; protein
log2 mRNA (strain/euploid)
log2 protein (strain/euploid)
chromosome
1 2 3 4 5 6 7 8 9 10 11 12 13 14 15 16
−2 −1 0 1 2

BML, 4n, aneu;−1*3;+1*6;−1*7;−1*10; mRNA

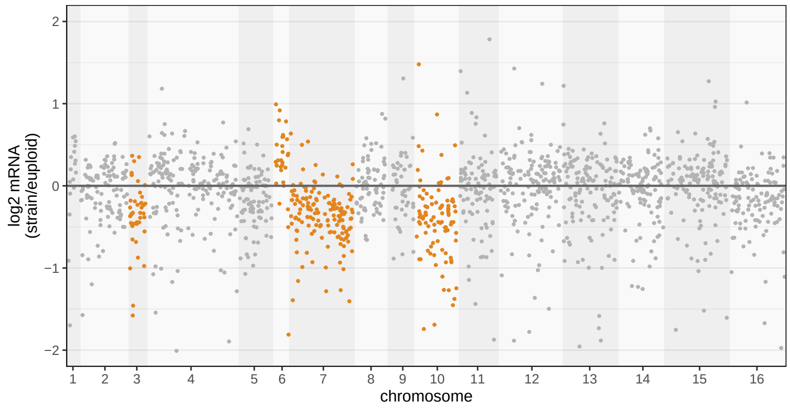


BML, 4n, aneu;−1*3;+1*6;−1*7;−1*10; protein

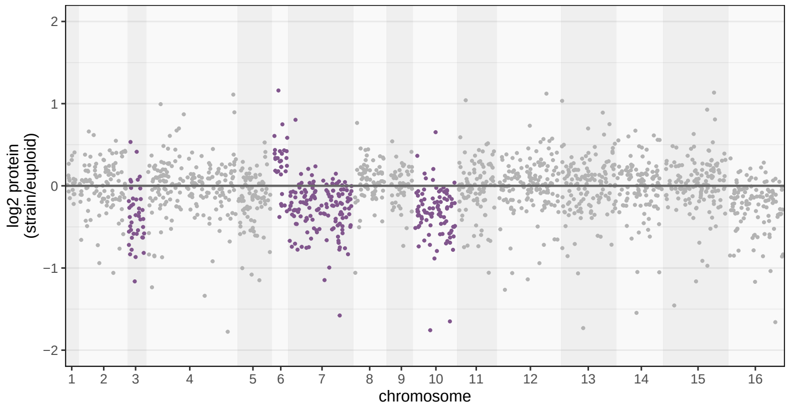


BMM, 4n, aneu;−1*1;−1*6;+1*7;−1*9;+1*13;+1*15; mRNA

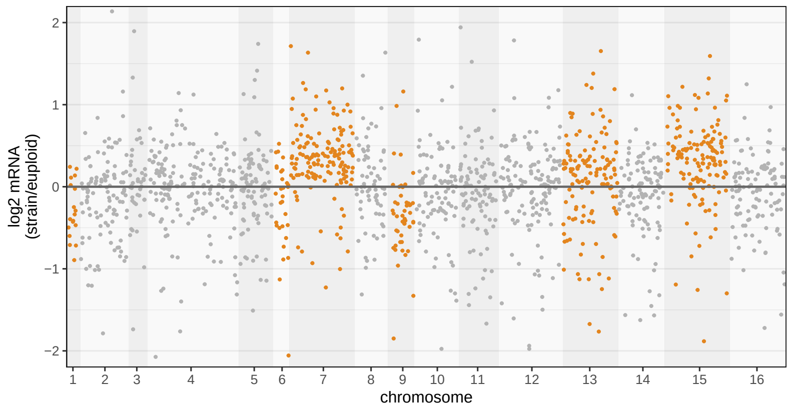


BMM, 4n, aneu;−1*1;−1*6;+1*7;−1*9;+1*13;+1*15; protein

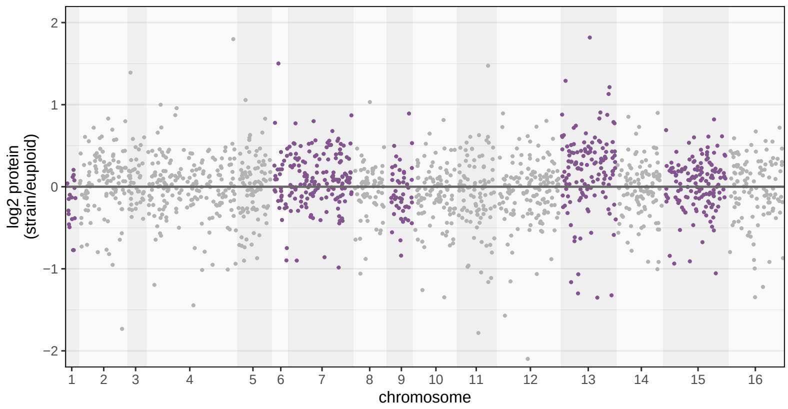


BMQ, 2n, aneu;+1*1;+1*6;+1*7;+1*9;+1*11;+1*12;+1*13; mRNA

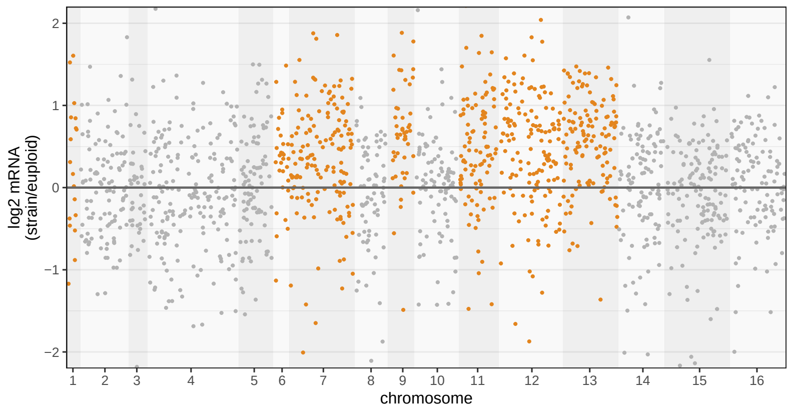


BMQ, 2n, aneu;+1*1;+1*6;+1*7;+1*9;+1*11;+1*12;+1*13; protein

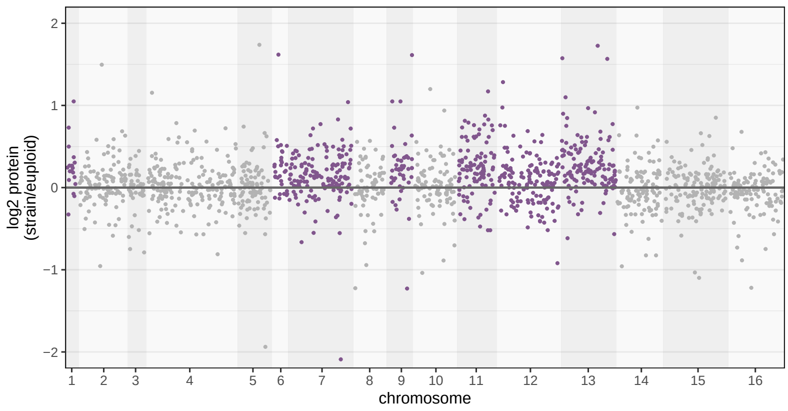


BPA, 2n, aneu;+1*8; mRNA

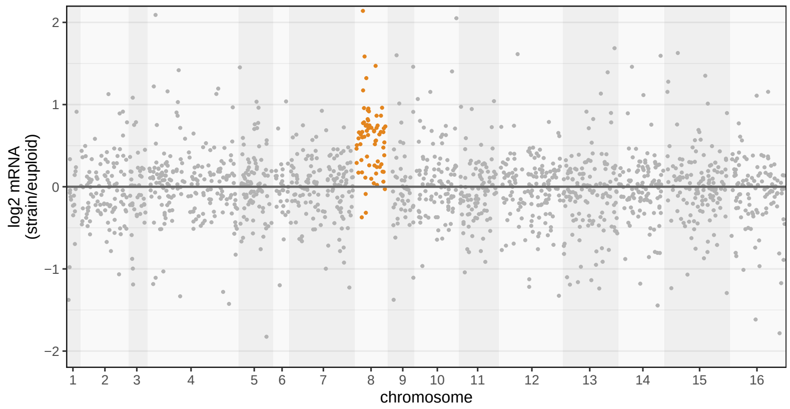


BPA, 2n, aneu;+1*8; protein

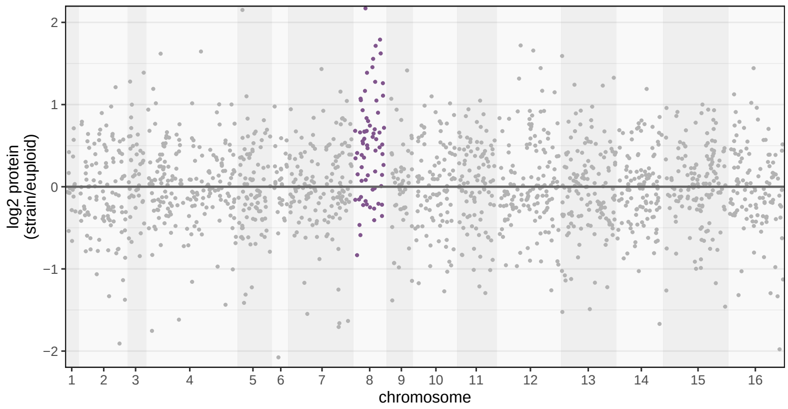


BPG, 2n, aneu;+1*1; mRNA

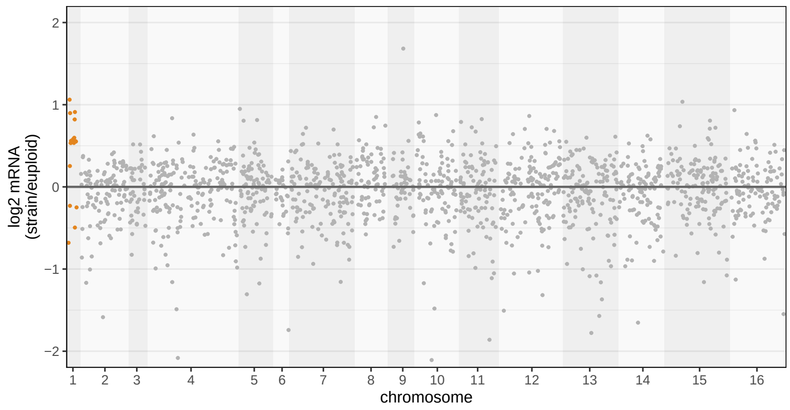


BPG, 2n, aneu;+1*1; protein

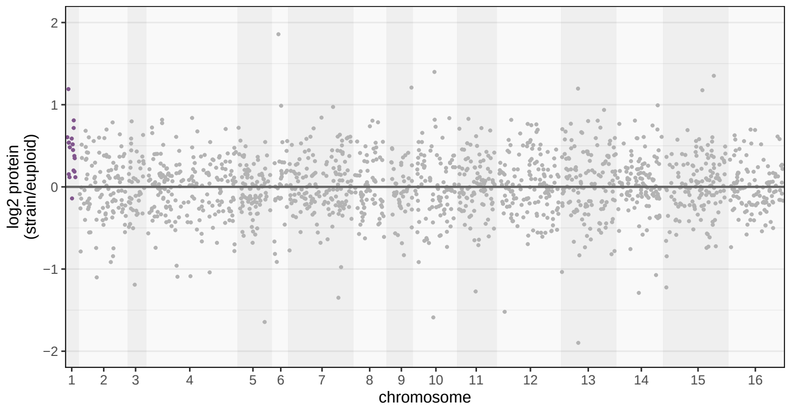

BPH, 2n, aneu;+1*1; mRNA

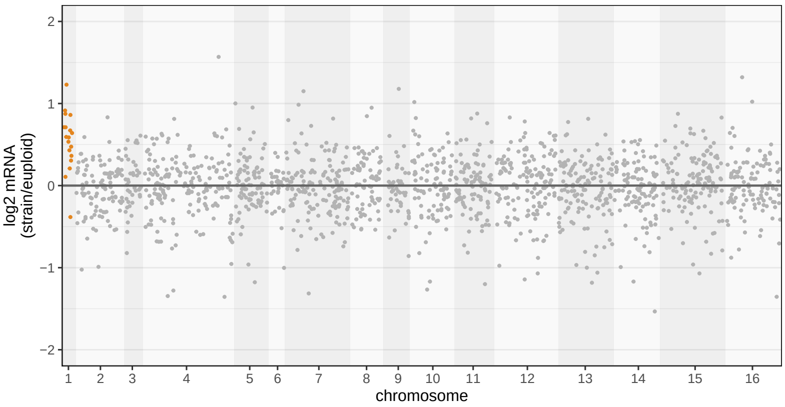


BPH, 2n, aneu;+1*1; protein

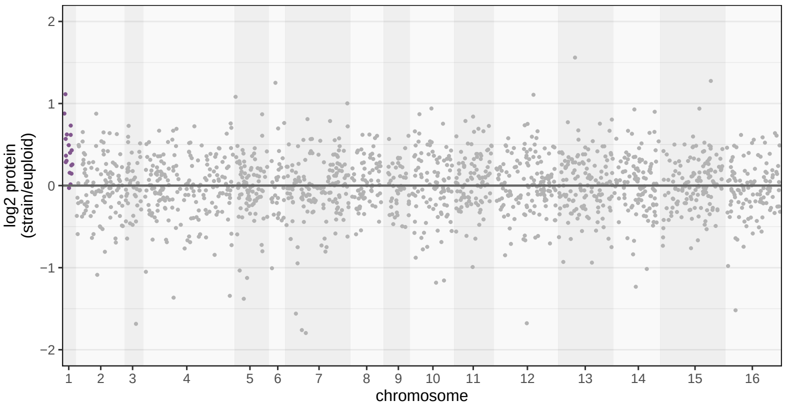


BPP, 2n, aneu;+1*1; mRNA

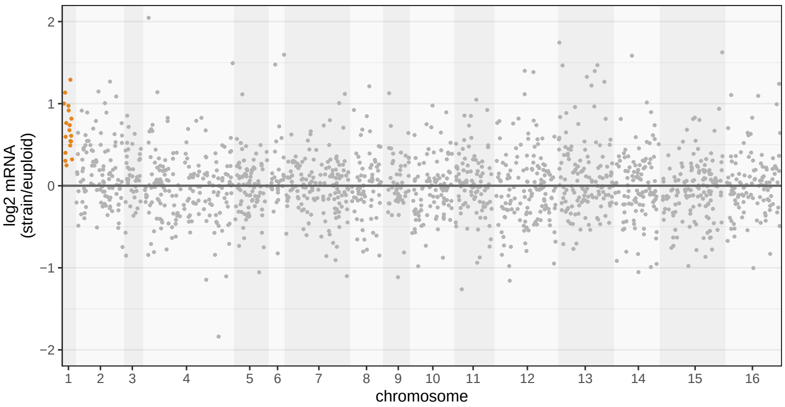


BPP, 2n, aneu;+1*1; protein

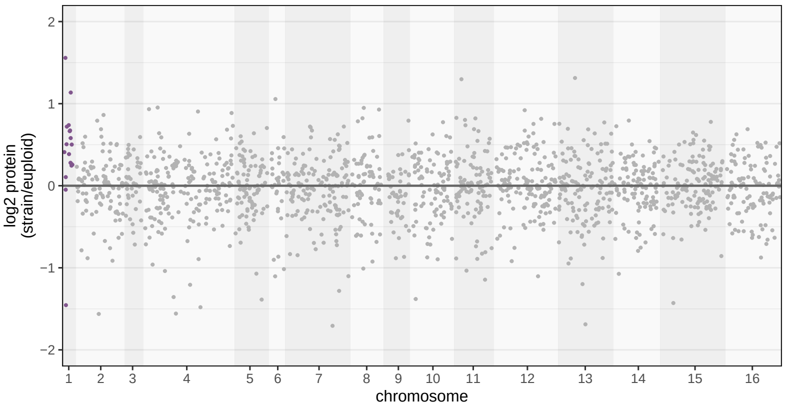


BRP, 4n, aneu;+1*1;−1*3; mRNA

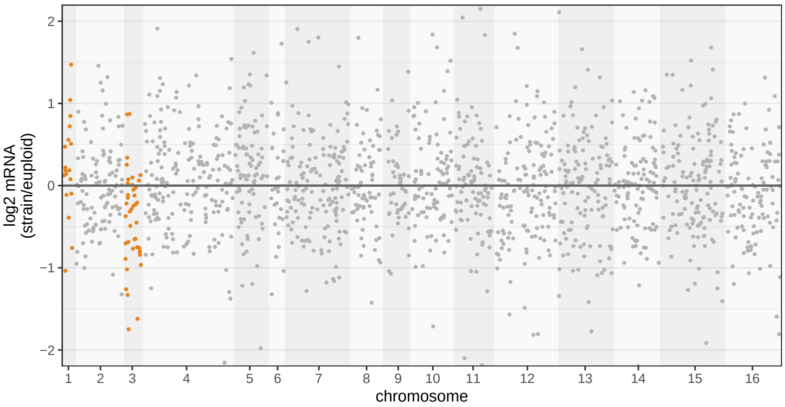


BRP, 4n, aneu;+1*1;−1*3; protein

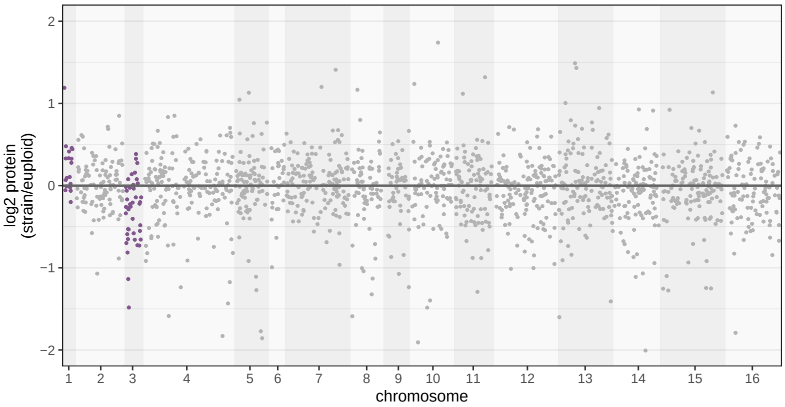


BTD, 4n, aneu;+1*10; mRNA

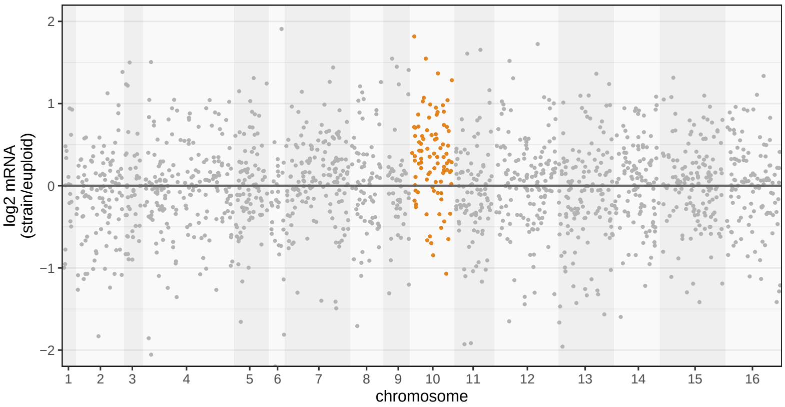


BTD, 4n, aneu;+1*10; protein

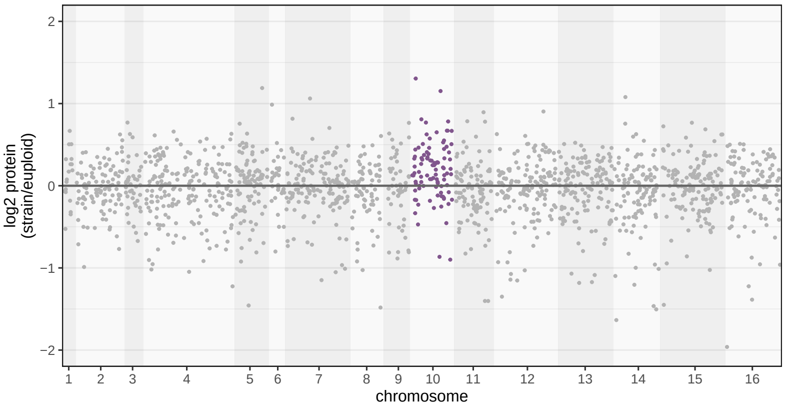


BTF, 2n, aneu;+1*1;+1*9; mRNA

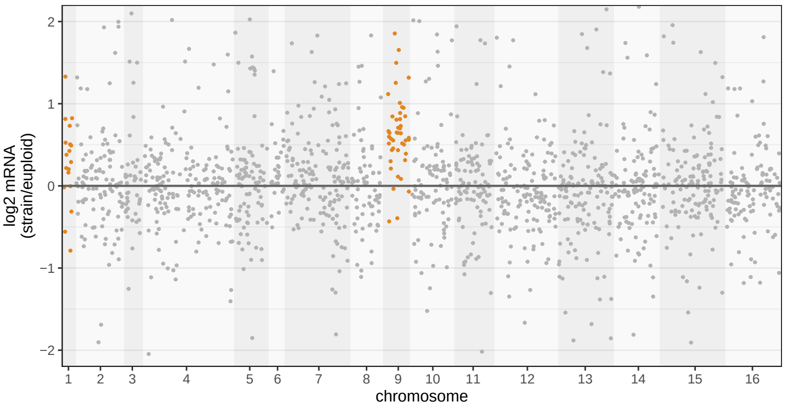


BTF, 2n, aneu;+1*1;+1*9; protein

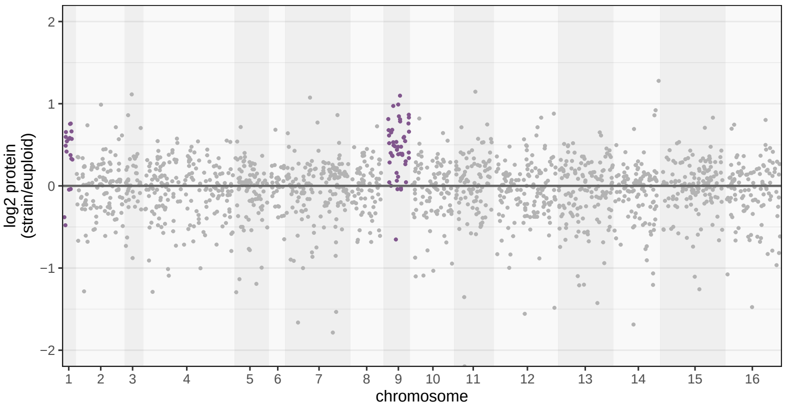

BTS, 2n, aneu;+1*11; mRNA

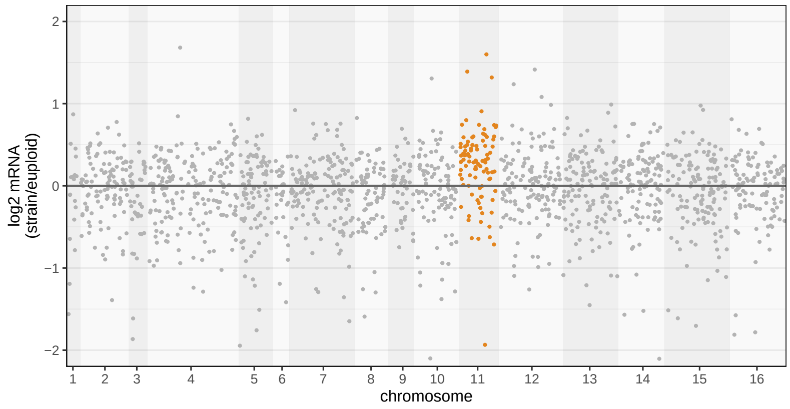


BTS, 2n, aneu;+1*11; protein

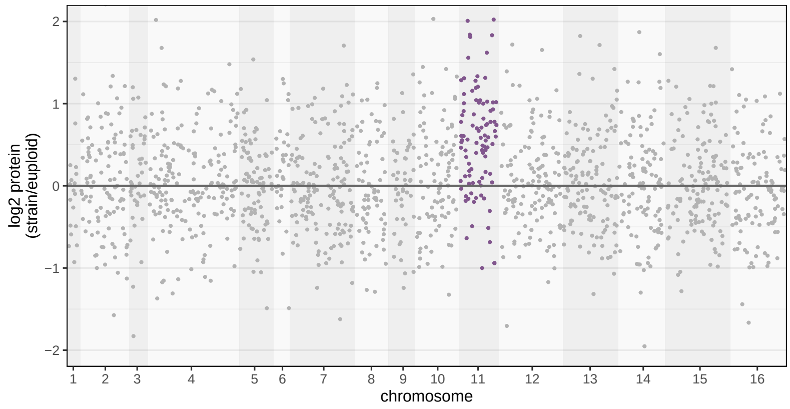


CAH, 2n, aneu;+1*4; mRNA

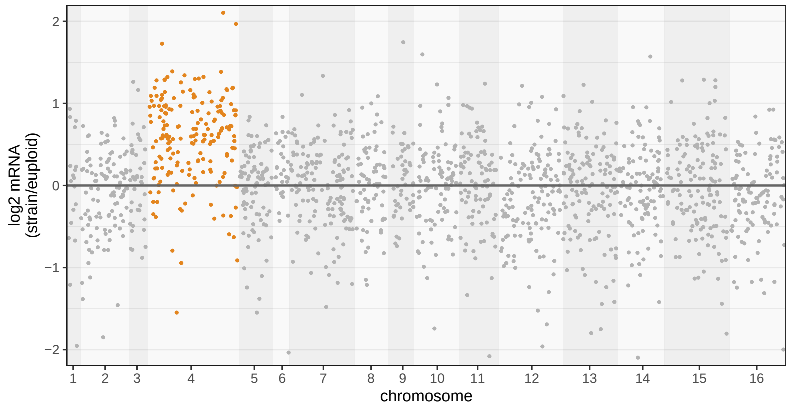


CAH, 2n, aneu;+1*4; protein

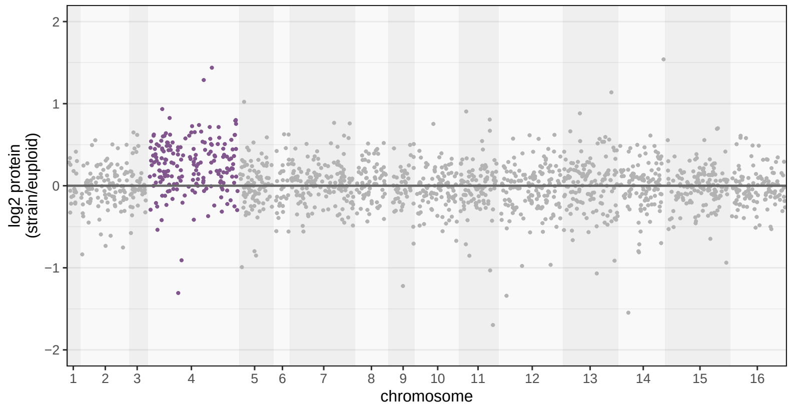


CAN, 2n, aneu;+1*9; mRNA

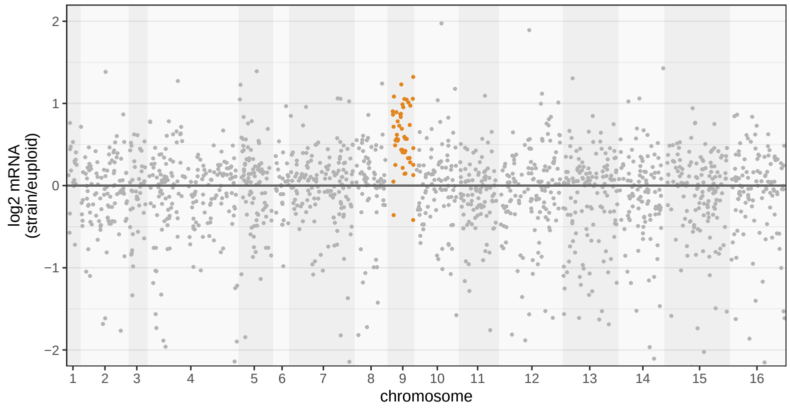


CAN, 2n, aneu;+1*9; protein

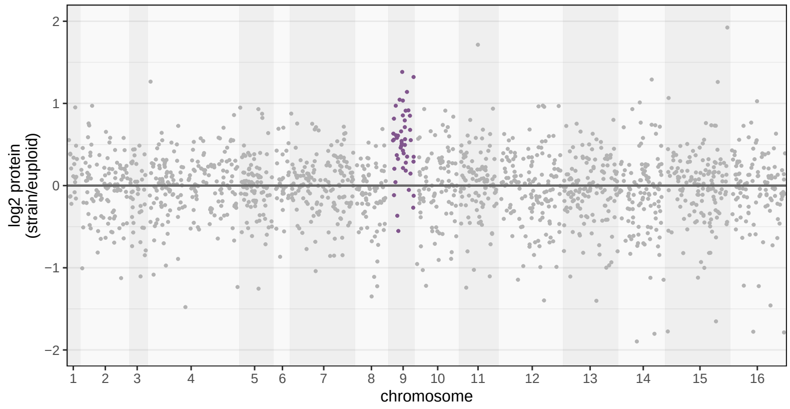


CCH, 2n, aneu;+1*16; mRNA

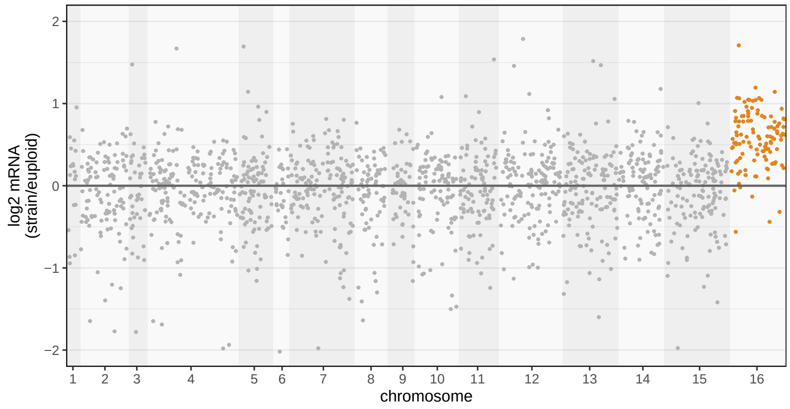


CCH, 2n, aneu;+1*16; protein

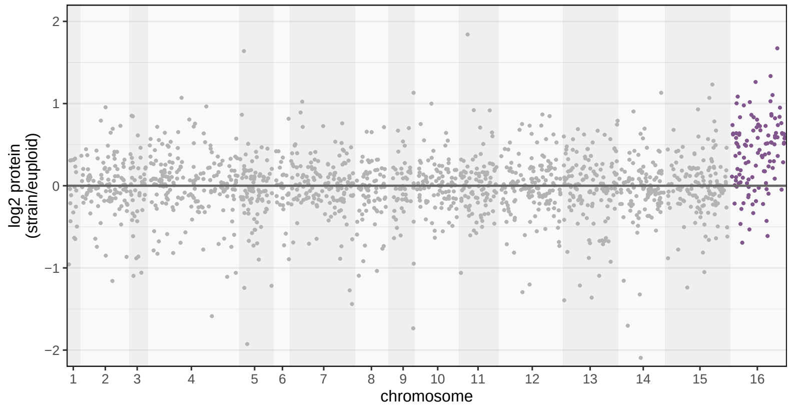


CDN, 2n, aneu;+2*1;+2*3; mRNA

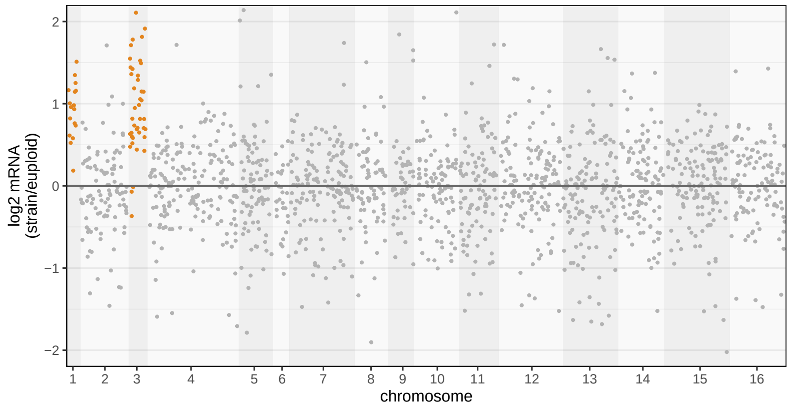


CDN, 2n, aneu;+2*1;+2*3; protein

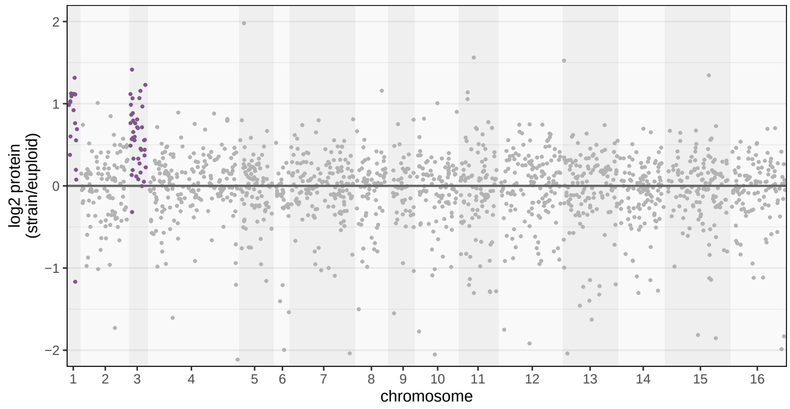

CFB, 2n, aneu;+2*9; mRNA

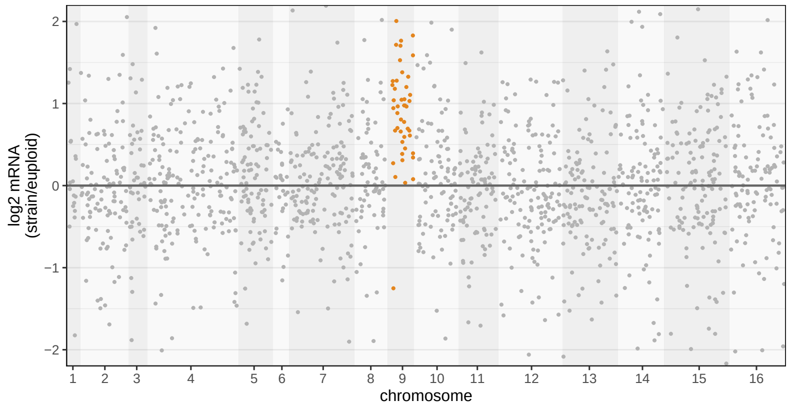


CFB, 2n, aneu;+2*9; protein

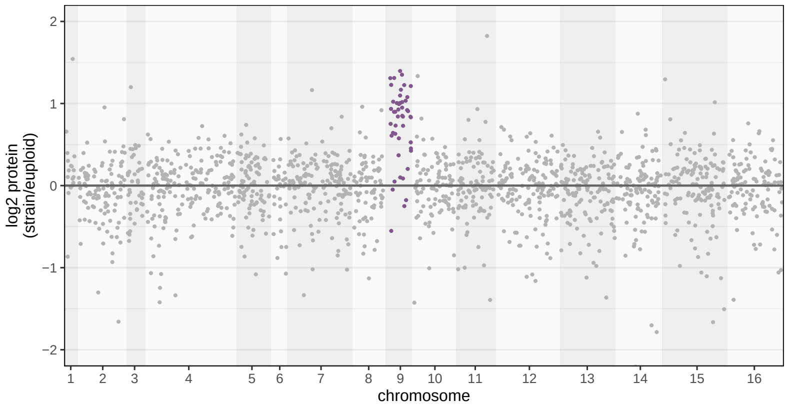


CFT, 2n, aneu;+1*5;+1*9; mRNA

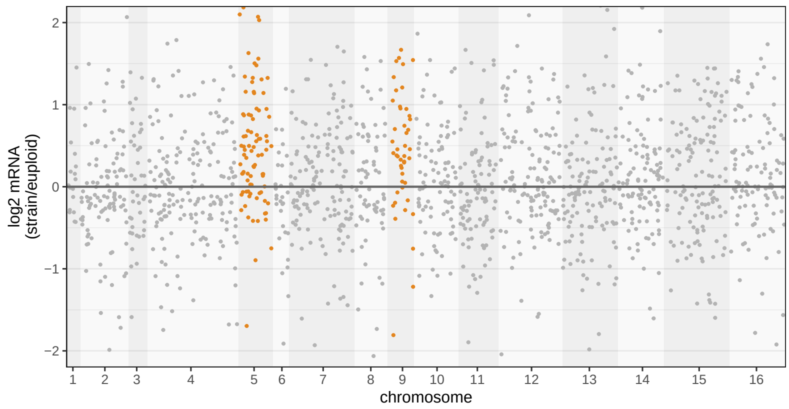


CFT, 2n, aneu;+1*5;+1*9; protein

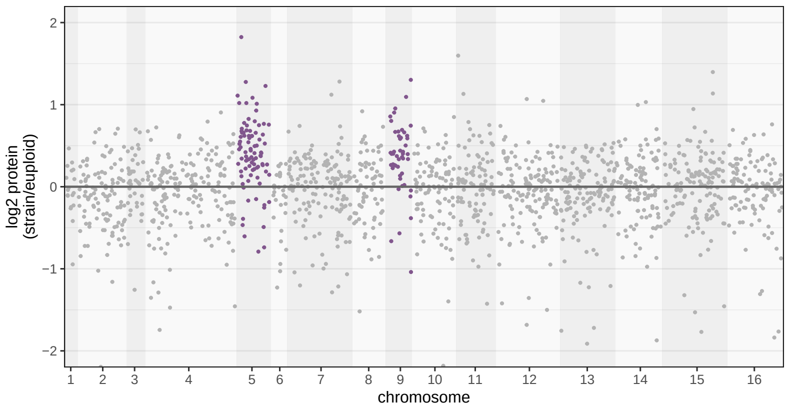


CFV, 2n, aneu;+1*1;+1*4; mRNA

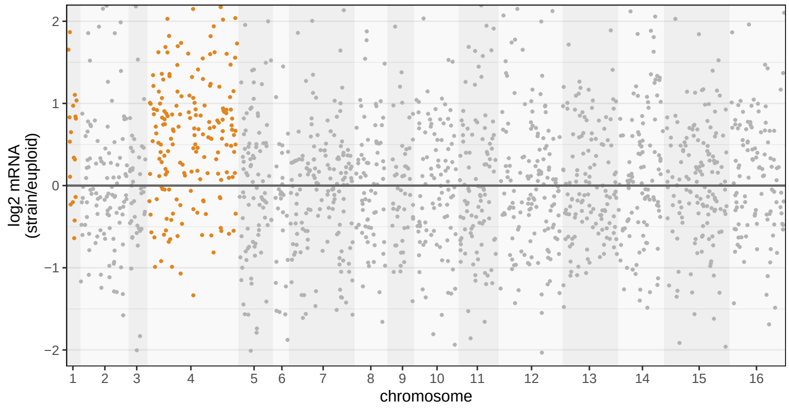


CFV, 2n, aneu;+1*1;+1*4; protein

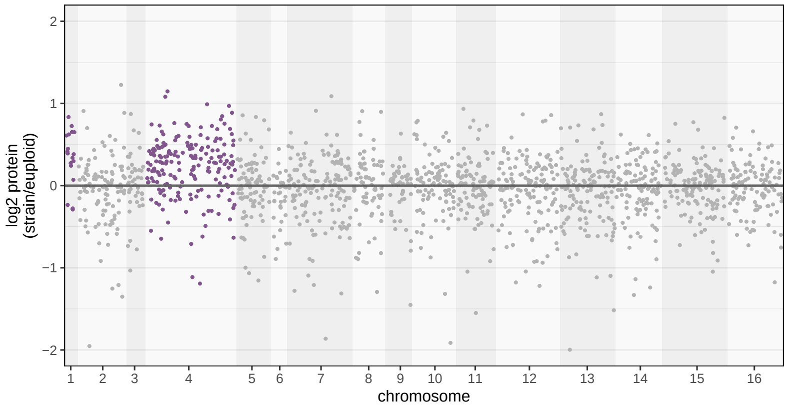


CHH, 2n, aneu;+1*7; mRNA

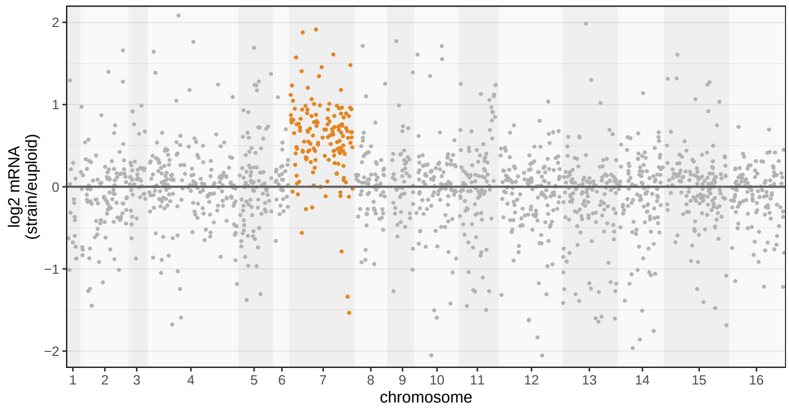


CHH, 2n, aneu;+1*7; protein

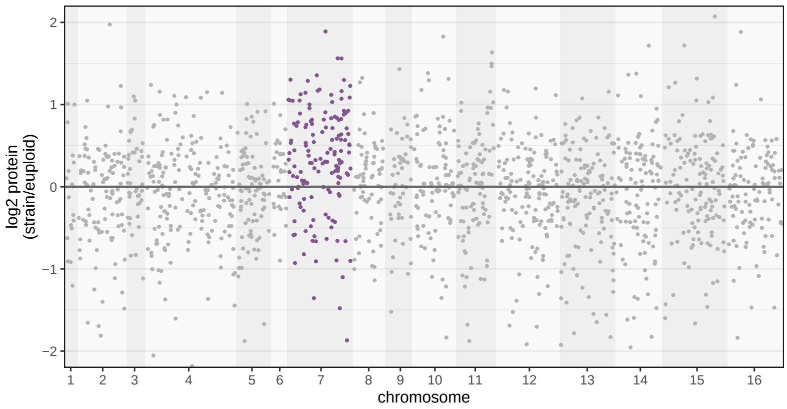


CIM, 2n, aneu;+2*9; mRNA

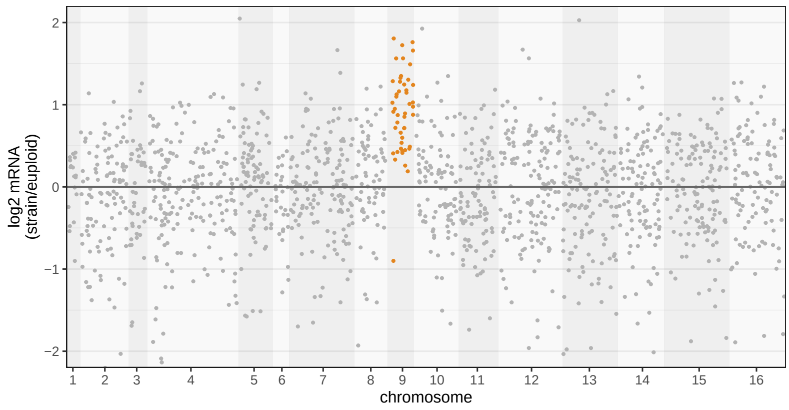


CIM, 2n, aneu;+2*9; protein

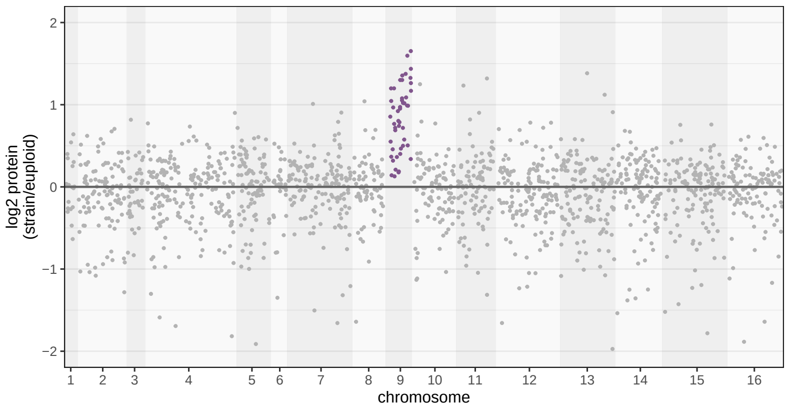

CKS, 4n, aneu;+1*15;−1*16; mRNA

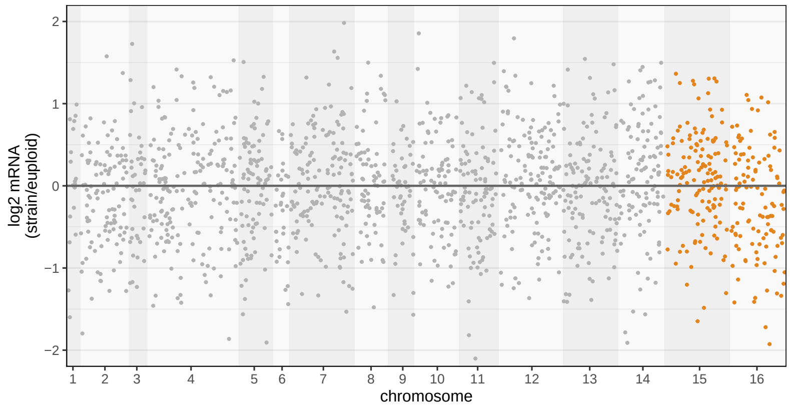


CKS, 4n, aneu;+1*15;−1*16; protein

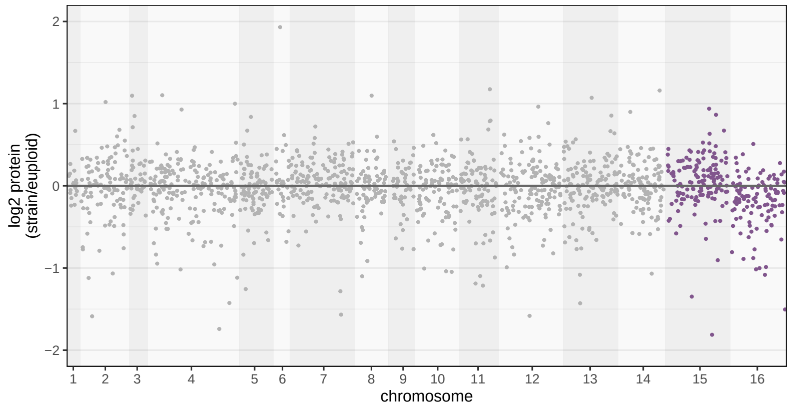


CLD, 2n, aneu;+2*3; mRNA

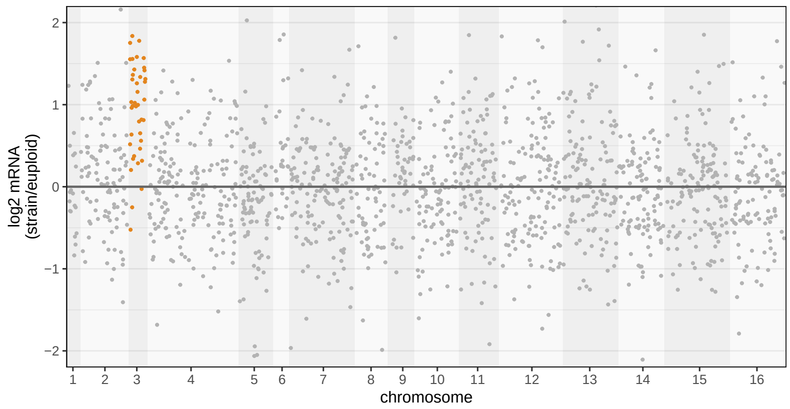


CLD, 2n, aneu;+2*3; protein

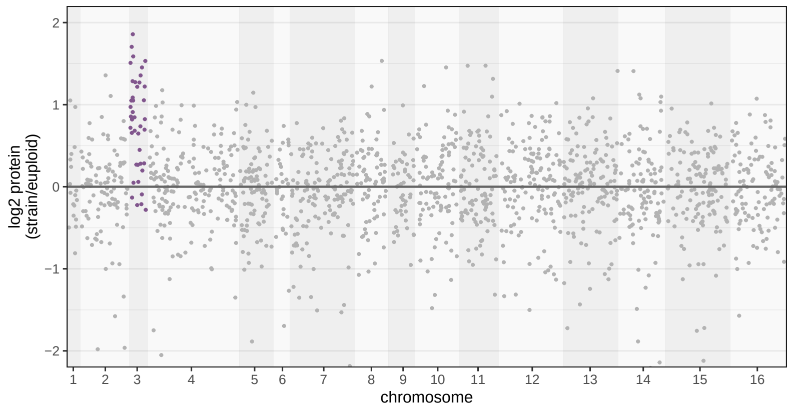


CLT, 2n, aneu;+1*9; mRNA

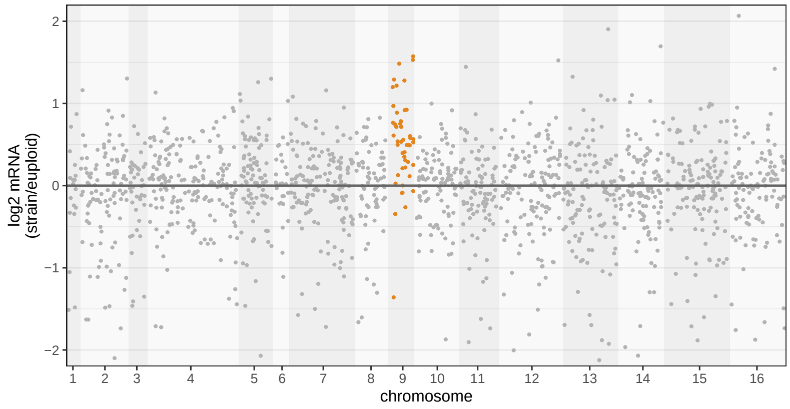


CLT, 2n, aneu;+1*9; protein

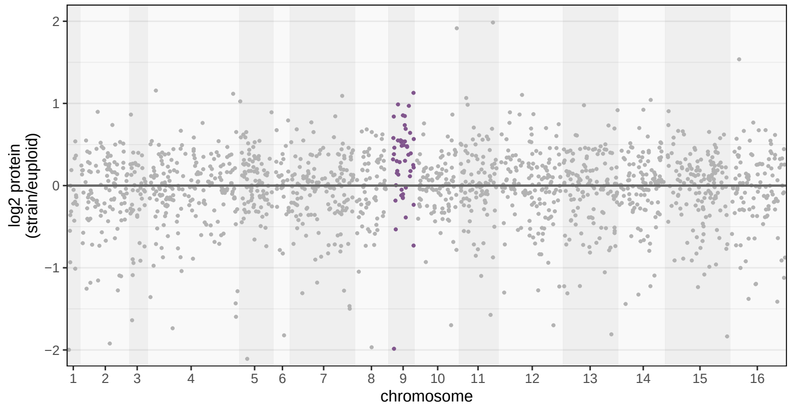


CMC, 2n, aneu;+1*14; mRNA

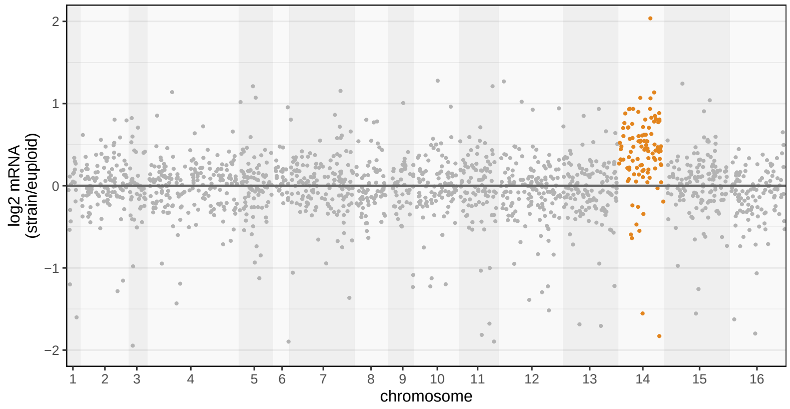


CMC, 2n, aneu;+1*14; protein

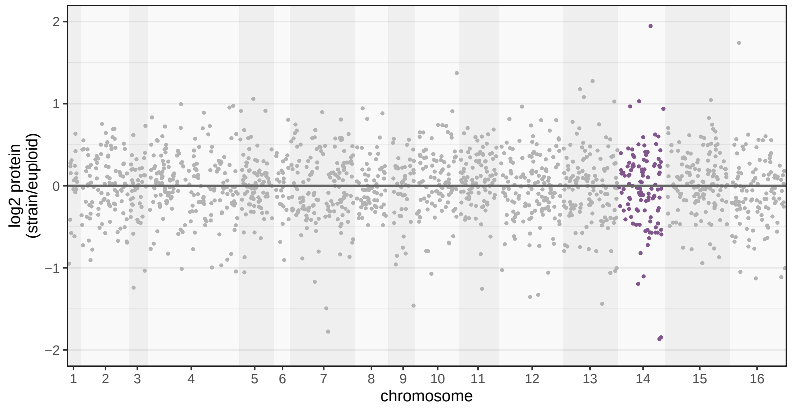


CMD, 2n, aneu;+1*3;+1*9; mRNA

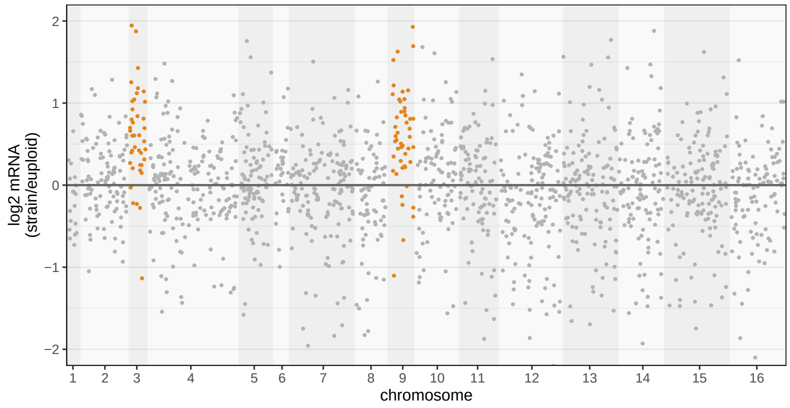


CMD, 2n, aneu;+1*3;+1*9; protein

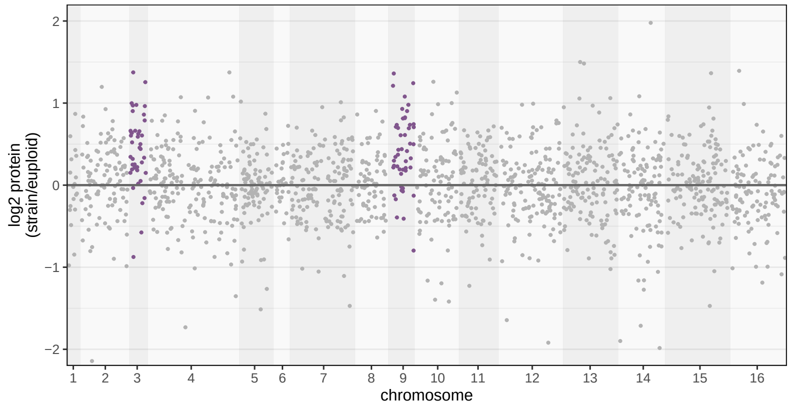

CME, 2n, aneu;+1*9; mRNA

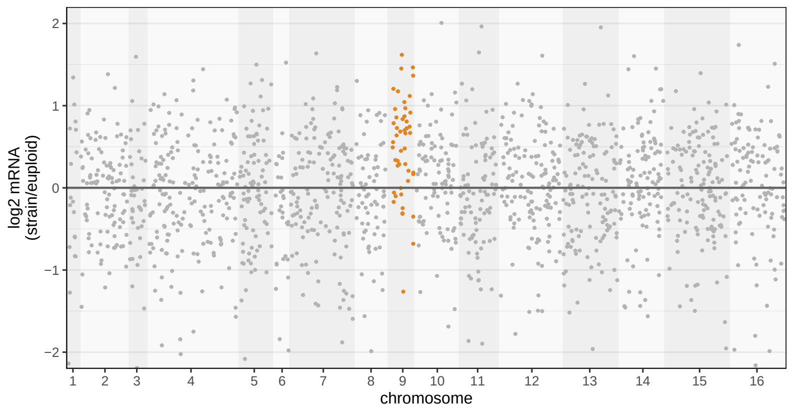


CME, 2n, aneu;+1*9; protein

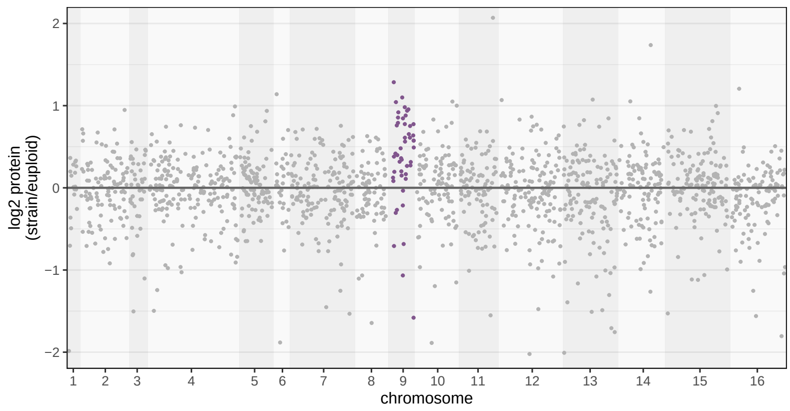


CMF, 2n, aneu;+1*3; mRNA

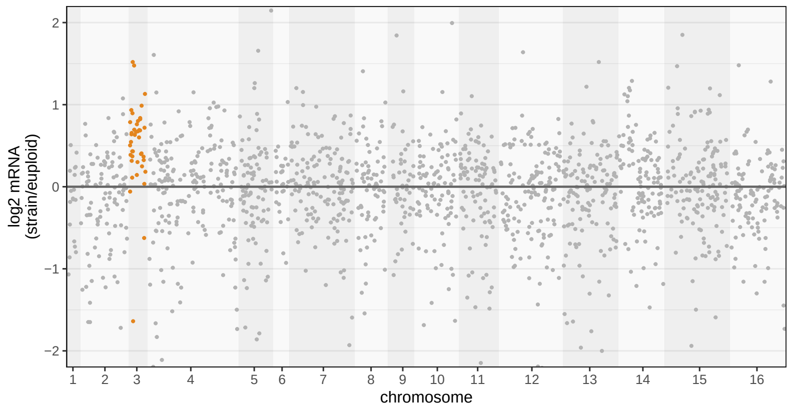


CMF, 2n, aneu;+1*3; protein

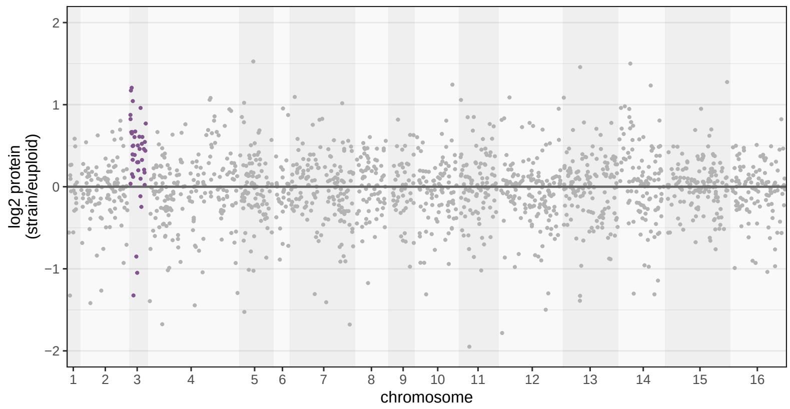


CML, 2n, aneu;−1*1; mRNA

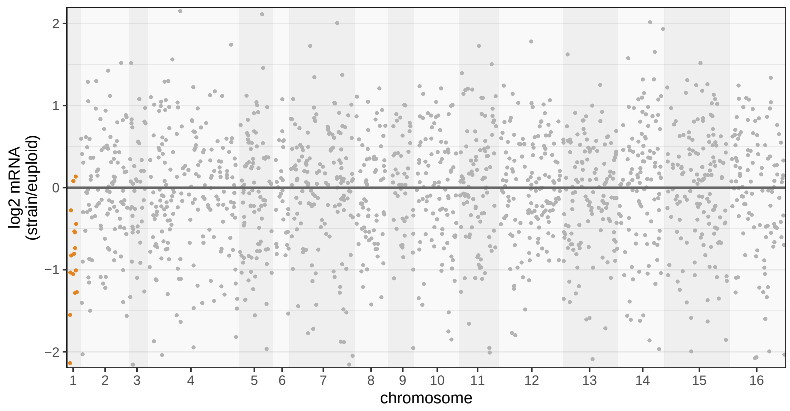


CML, 2n, aneu;−1*1; protein

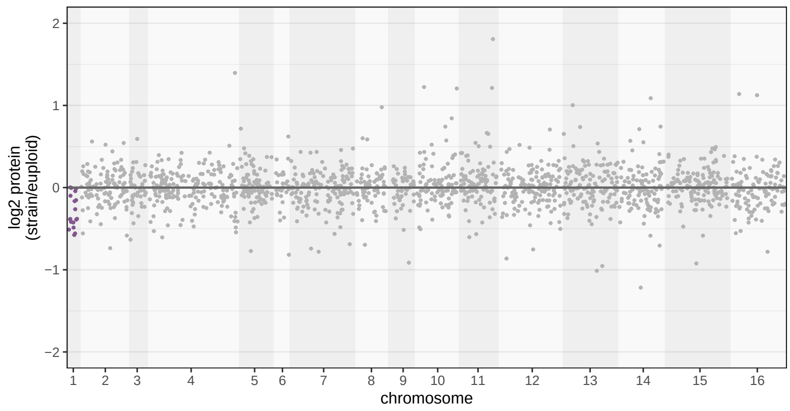


CMM, 2n, aneu;+1*3; mRNA

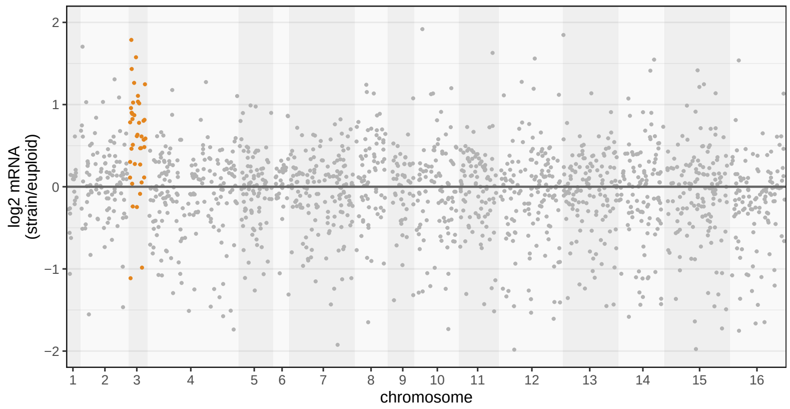


CMM, 2n, aneu;+1*3; protein

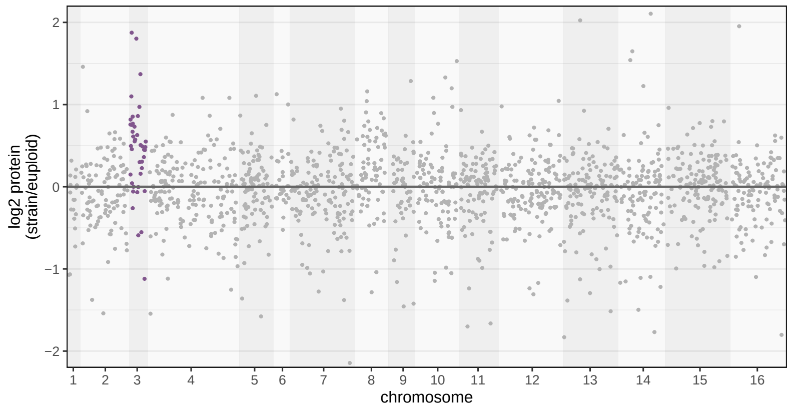


CMN, 2n, aneu;+1*1; mRNA

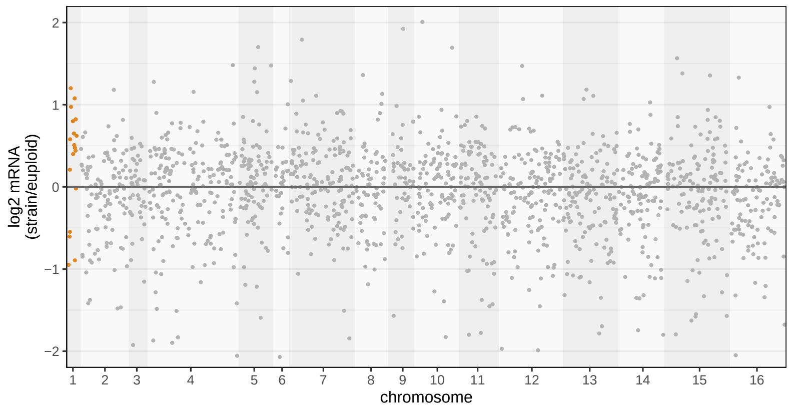


CMN, 2n, aneu;+1*1; protein

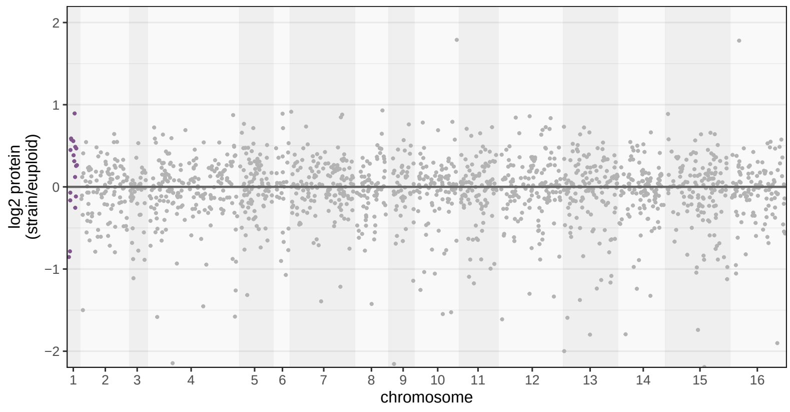

CNL, 2n, aneu;+1*3; mRNA

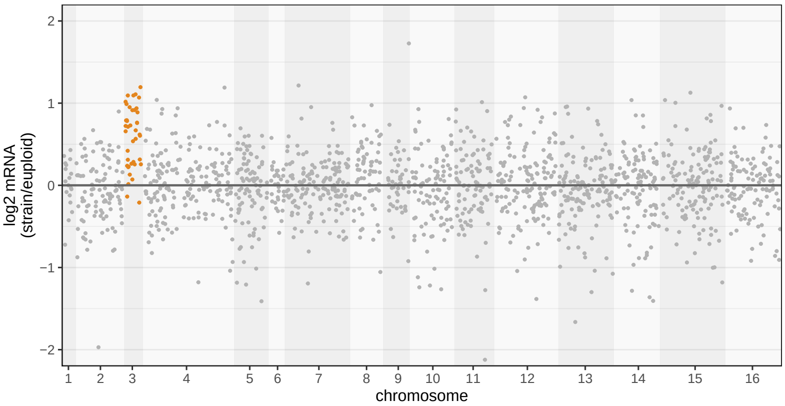


CNL, 2n, aneu;+1*3; protein

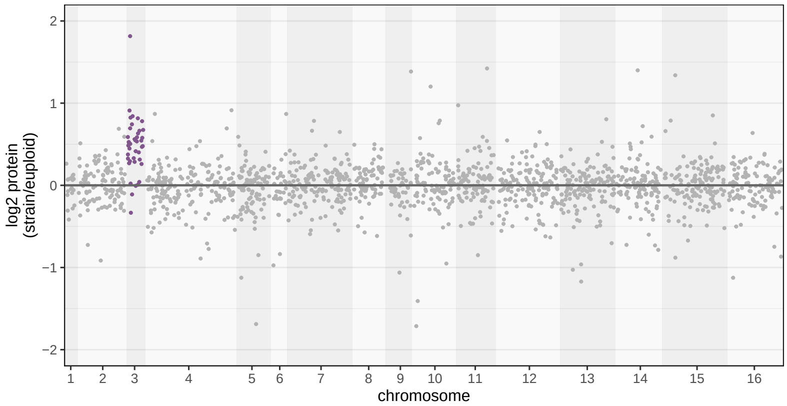


CPB, 2n, aneu;+1*3; mRNA

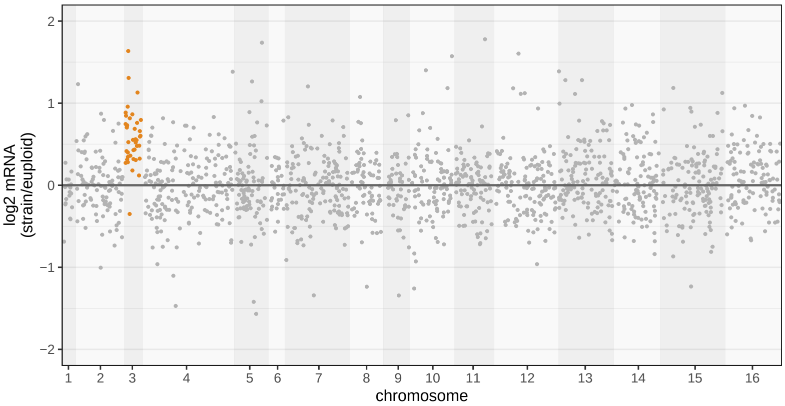


CPB, 2n, aneu;+1*3; protein

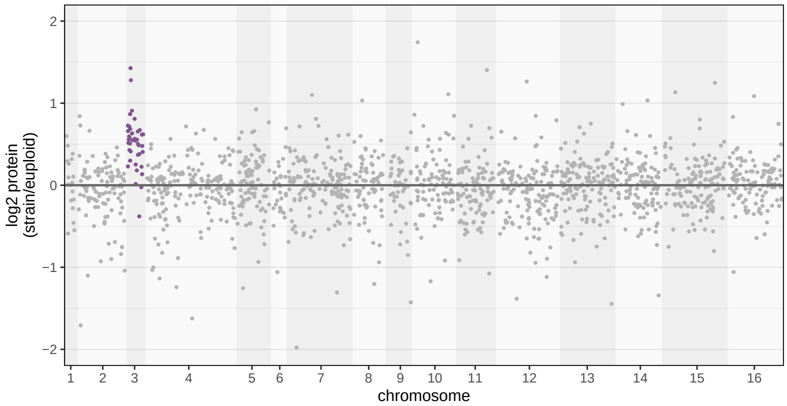


CPE, 2n, aneu;+1*1; mRNA

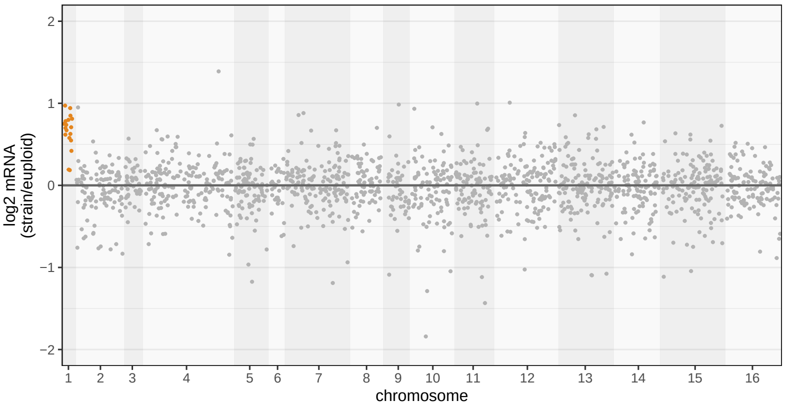


CPE, 2n, aneu;+1*1; protein

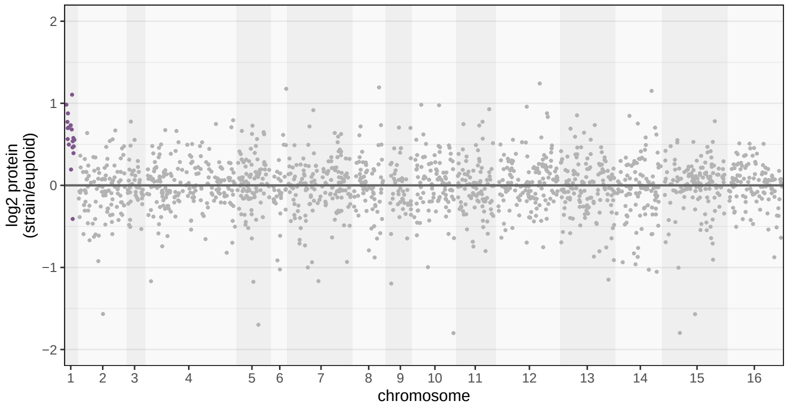


CPQ, 2n, aneu;+1*11; mRNA

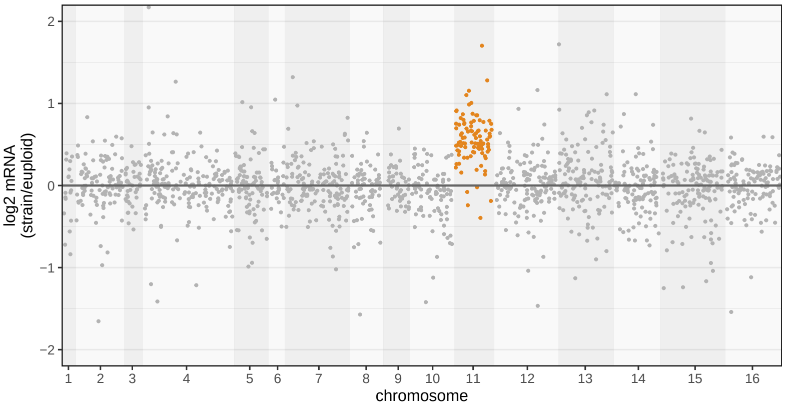


CPQ, 2n, aneu;+1*11; protein

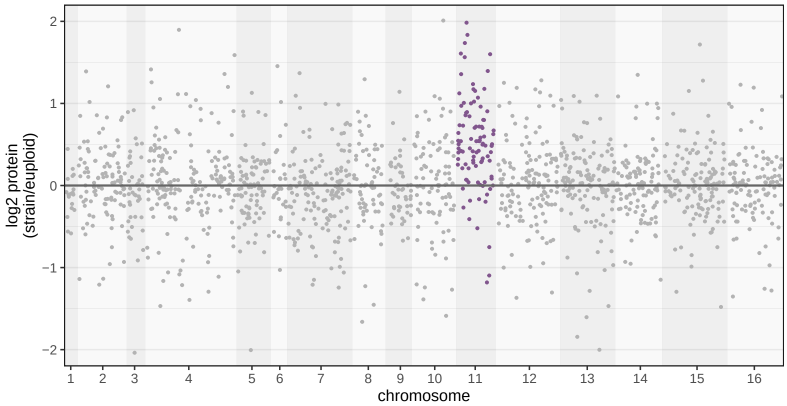


CPR, 2n, aneu;+1*12; mRNA

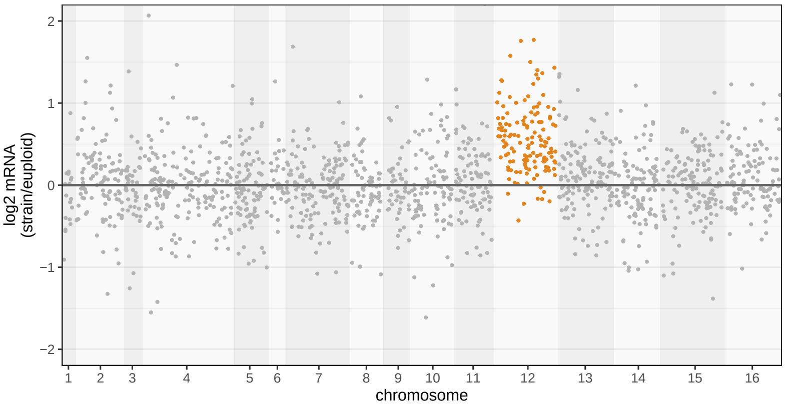


CPR, 2n, aneu;+1*12; protein

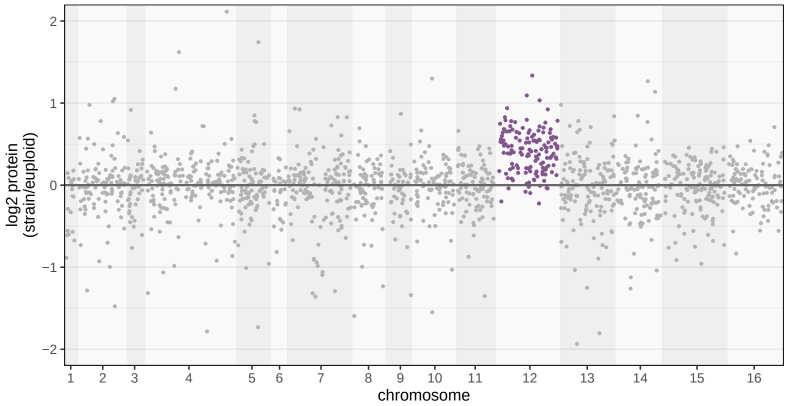

CPT, 2n, aneu;+1*9; mRNA

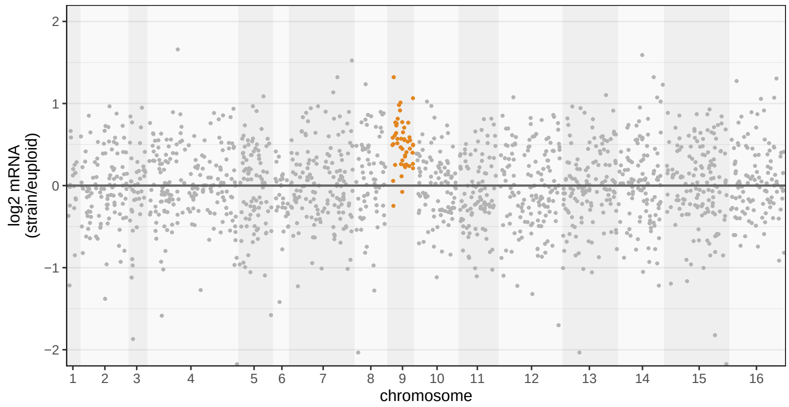


CPT, 2n, aneu;+1*9; protein

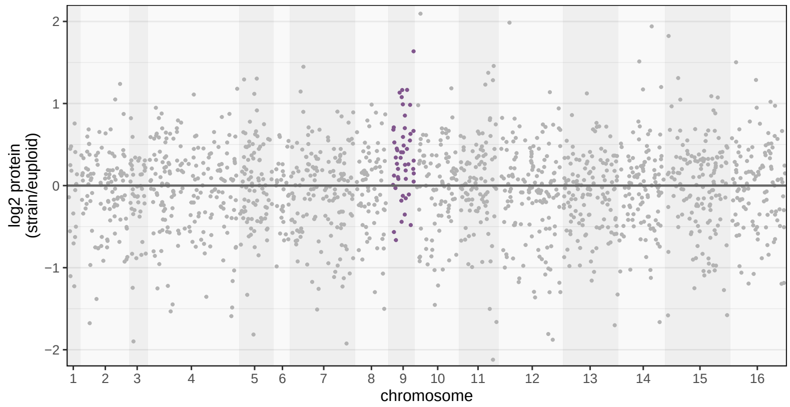


CQQ, 2n, aneu;+1*3; mRNA

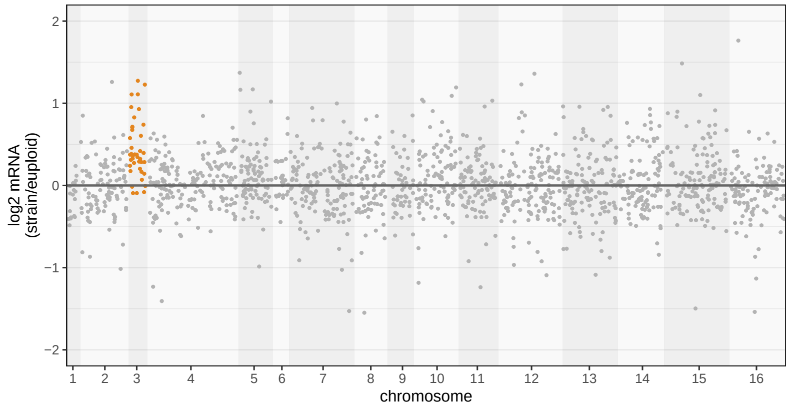


CQQ, 2n, aneu;+1*3; protein

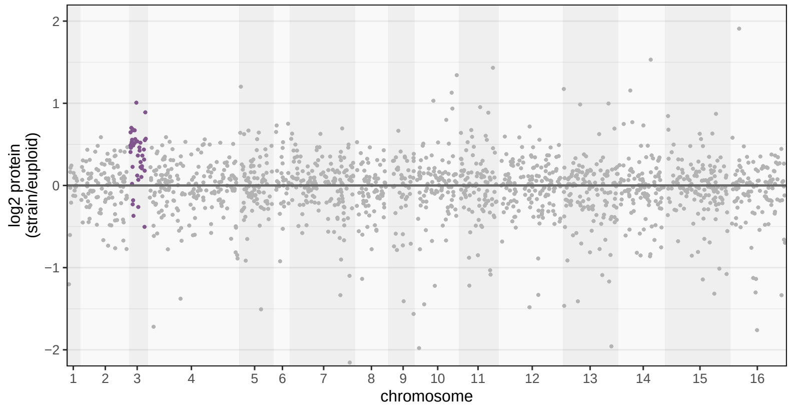


CQR, 2n, aneu;+1*3; mRNA

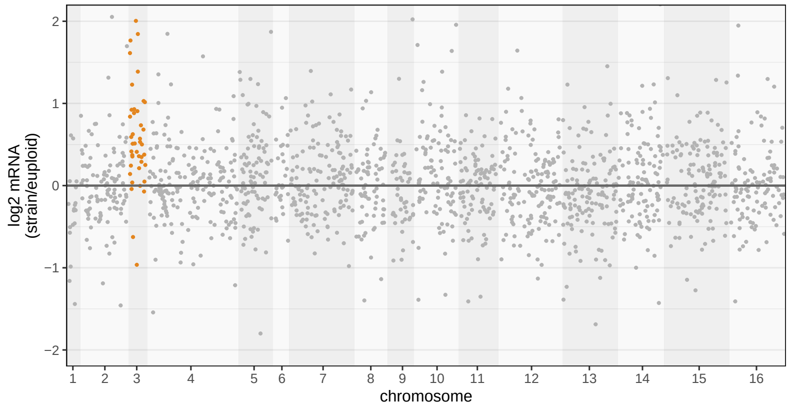


CQR, 2n, aneu;+1*3; protein

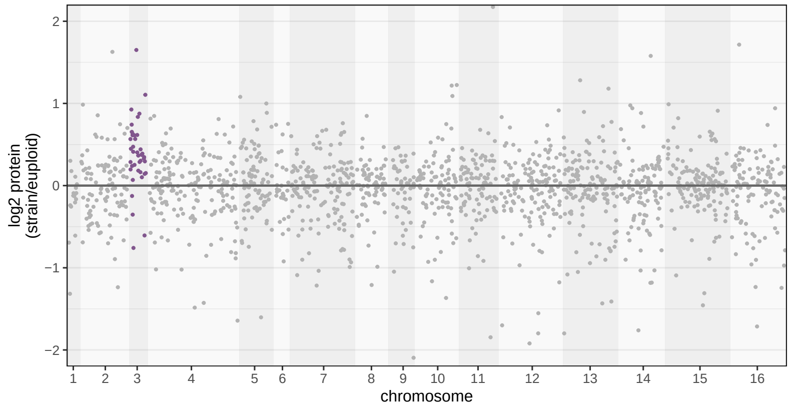


CRA, 2n, aneu;−1*1; mRNA

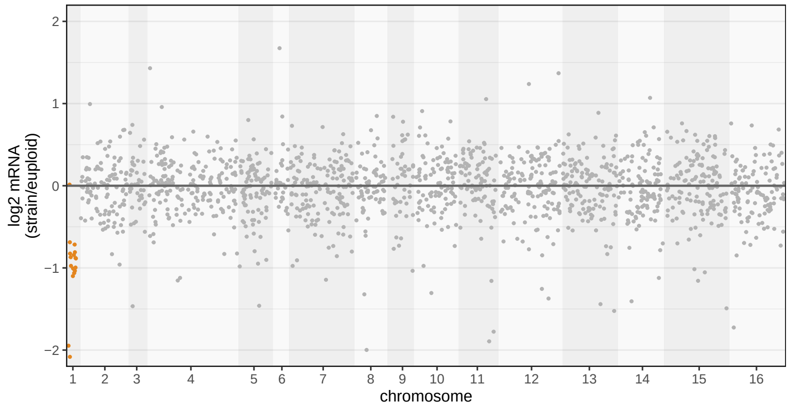


CRA, 2n, aneu;−1*1; protein

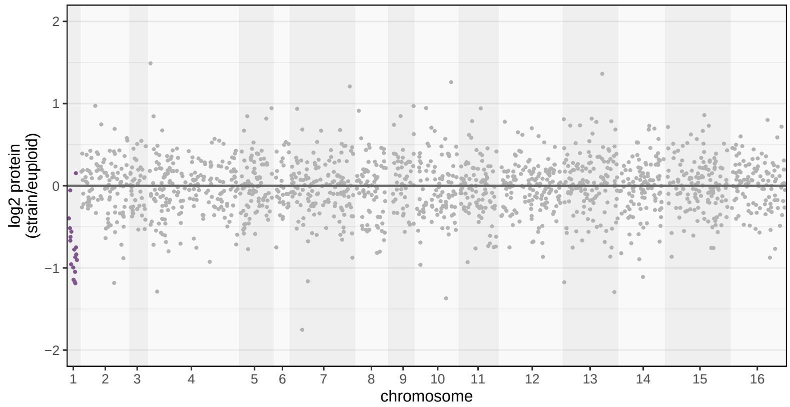


CRI, 2n, aneu;+1*4;+1*8;+1*15; mRNA

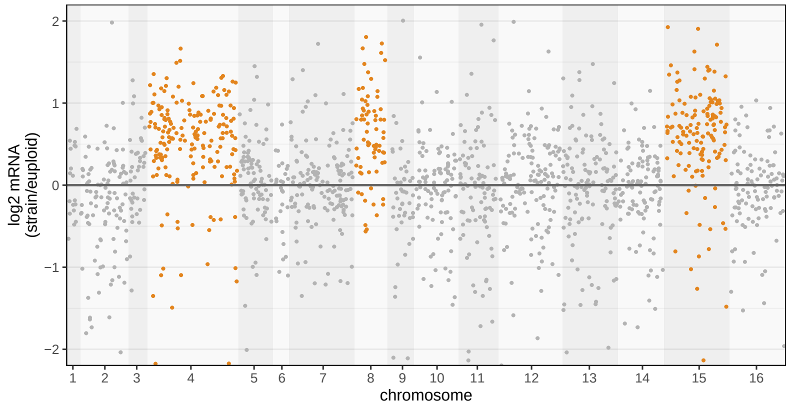


CRI, 2n, aneu;+1*4;+1*8;+1*15; protein

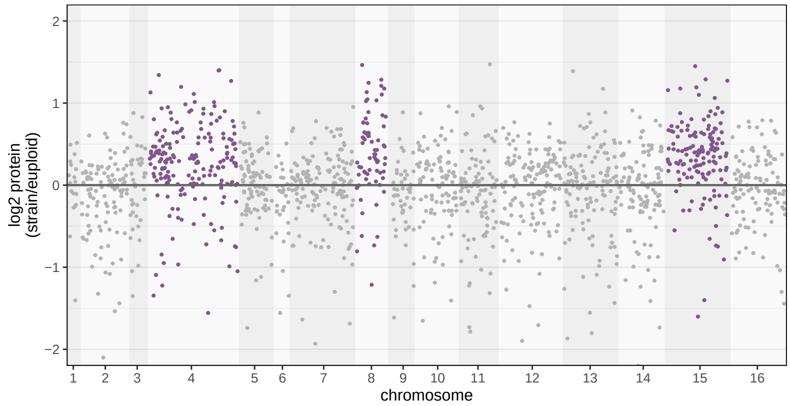

**Supplementary Fig. 2**, page 22: High-resolution version of the heatmap displayed in Fig. 4a including isolate names.

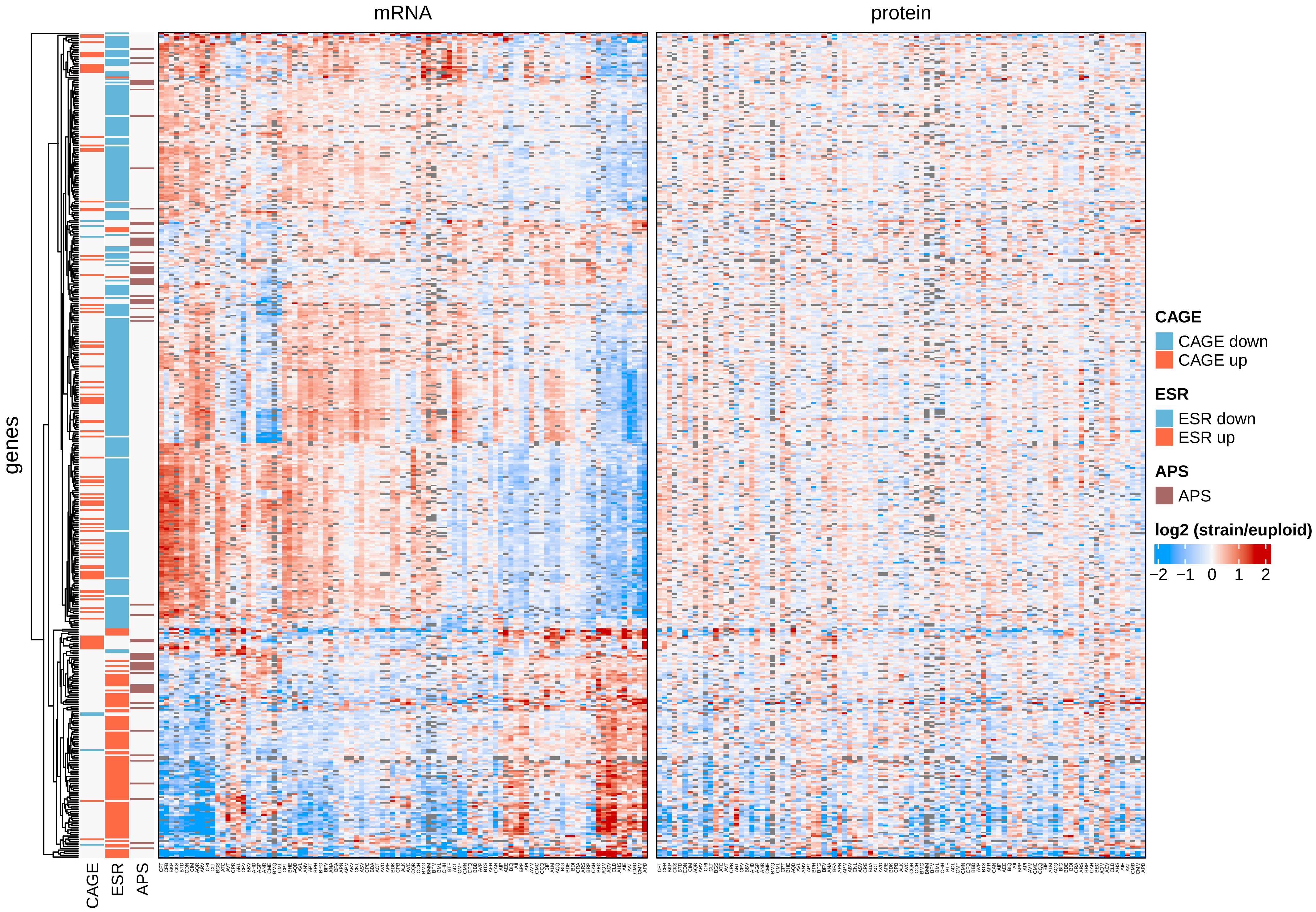

mRNA
protein
genes
CAGE
ESR
APS
CAGE
CAGE down
CAGE up
ESR
ESR down
ESR up
APS
APS
log2 (strain/euploid)
−2 −1 0 1 2
