# Supplementary material for: Natural proteome diversity links aneuploidy tolerance to protein turnover
Source: Nature. 2024 May 22;630(8015):149–57. doi: 10.1038/s41586-024-07442-9 (PMC11153158; doi:10.1038/s41586-024-07442-9)
Supplement: Supplementary file 2 — Reporting Summary [file 41586_2024_7442_MOESM2_ESM.pdf]

nature portfolio

Corresponding author(s): Prof. Dr Markus Ralser

Last updated by author(s): Mar 19, 2024

Reporting Summary

Nature Portfolio wishes to improve the reproducibility of the work that we publish. This form provides structure for consistency and transparency in reporting. For further information on Nature Portfolio policies, see our Editorial Policies and the Editorial Policy Checklist.

Statistics

For all statistical analyses, confirm that the following items are present in the figure legend, table legend, main text, or Methods section.

n/a	Confirmed	
☐	☒	The exact sample size (*n*) for each experimental group/condition, given as a discrete number and unit of measurement
☐	☒	A statement on whether measurements were taken from distinct samples or whether the same sample was measured repeatedly
☐	☒	The statistical test(s) used AND whether they are one- or two-sided<br>*Only common tests should be described solely by name; describe more complex techniques in the Methods section.*
☒	☐	A description of all covariates tested
☐	☒	A description of any assumptions or corrections, such as tests of normality and adjustment for multiple comparisons
☐	☒	A full description of the statistical parameters including central tendency (e.g. means) or other basic estimates (e.g. regression coefficient) AND variation (e.g. standard deviation) or associated estimates of uncertainty (e.g. confidence intervals)
☐	☒	For null hypothesis testing, the test statistic (e.g. *F*, *t*, *r*) with confidence intervals, effect sizes, degrees of freedom and *P* value noted<br>*Give P values as exact values whenever suitable.*
☒	☐	For Bayesian analysis, information on the choice of priors and Markov chain Monte Carlo settings
☒	☐	For hierarchical and complex designs, identification of the appropriate level for tests and full reporting of outcomes
☒	☐	Estimates of effect sizes (e.g. Cohen's *d*, Pearson's *r*), indicating how they were calculated

Our web collection on statistics for biologists contains articles on many of the points above.

Software and code

Policy information about availability of computer code

Data collection	Proteomic raw data were collected using Analyst 1.8.1. Metabolomic raw data were collected using Mass Hunter B.07.01.
Data analysis	Raw mass spectrometric data for natural isolates and disomic strains were processed using DIA-NN version 1.7.12. Ubiquitinomics data and time course proteomics data were processed using DIA-NN version 1.8. Raw mass spectrometric data of turnover samples were processed using MaxQuant version 1.6.7.0. Pre-processing and statistical analyses were performed using R 3.6. The following dedicated packages were used during analysis: diann 1.0.1, rstatix 0.7.2, data.table 1.14.2, org.Sc.sgd.db 3.18.0, ComplexHeatmap 2.18.0, pROC 1.18.5, edgeR 3.38.4.

For manuscripts utilizing custom algorithms or software that are central to the research but not yet described in published literature, software must be made available to editors and reviewers. We strongly encourage code deposition in a community repository (e.g. GitHub). See the Nature Portfolio guidelines for submitting code & software for further information.

Data

Policy information about availability of data

All manuscripts must include a data availability statement. This statement should provide the following information, where applicable:

- Accession codes, unique identifiers, or web links for publicly available datasets
- A description of any restrictions on data availability
- For clinical datasets or third party data, please ensure that the statement adheres to our policy

Raw and processed mass spectrometry data of the natural collection are deposited in Massive under ID MSV000090435. Data associated with the disomes

collection are deposited at the ProteomeXchange Consortium via the PRIDE partner repository with the dataset identifier PXD044526. Data associated with turnover experiments are deposited at the ProteomeXchange Consortium via PRIDE with the identifier PXD048219. The RNAseq data are available in the European Nucleotide Archive (ENA) under the accession number PRJEB52153. Further, the following publicly deposited data sources have been used: gene copy number annotation for natural isolates from the 1002 yeast genome website (http://1002genomes.u-strasbg.fr/files/); microarray gene expression data for lab-engineered disomic strains from doi: 10.1126/science.1142210; SILAC proteome data for lab-engineered disomic strains from doi: 10.7554/eLife.03023; proteomes of the aneuploid yeast deletion collection from doi: 10.1016/j.cell.2023.03.026 (https://y5k.bio.ed.ac.uk/); gene ontology annotation from SGD (https://www.yeastgenome.org/); yeast reference proteome databases from UniProt (https://www.uniprot.org); KEGG annotation from KEGG (https://www.genome.jp/kegg/); protein:protein interaction data from STRING (https://string-db.org); prediction of protein disorder and linear interacting peptides by alphafold, mobidb, and anchor from MobiDB (https://mobidb.bio.unipd.it/); ribosome occupancy data from doi: 10.1101/gr.164996.113; amino acid synthesis costs and glucose cost from doi: 10.1073/pnas.2114622119; absolute protein copy numbers per cell from doi: 10.1038/nature02046; protein half life data from doi: 10.1016/j.cels.2017.08.008; exponential degradation annotation from doi: 10.1016/j.cell.2016.09.015; protein complex membership annotation from the Complex Portal of the EBI (https://www.ebi.ac.uk/complexportal/home); annotation of ESR genes from doi: 10.1091/mbc.11.12.4241; annotation of CAGE genes from doi: 10.1038/s41586-019-1187-2; annotation of APS genes from doi: 10.7554/eLife.03023; proteasome component annotations from 10.1534/genetics.112.140467; chaperone system annotations from doi: 10.1016/j.celrep.2017.08.074.

Research involving human participants, their data, or biological material

Policy information about studies with human participants or human data. See also policy information about sex, gender (identity/presentation), and sexual orientation and race, ethnicity and racism.

Reporting on sex and gender	-NA
Reporting on race, ethnicity, or other socially relevant groupings	-NA
Population characteristics	-NA
Recruitment	-NA
Ethics oversight	-NA

Note that full information on the approval of the study protocol must also be provided in the manuscript.

Field-specific reporting

Please select the one below that is the best fit for your research. If you are not sure, read the appropriate sections before making your selection.

☒ Life sciences ☐ Behavioural & social sciences ☐ Ecological, evolutionary & environmental sciences

For a reference copy of the document with all sections, see nature.com/documents/nr-reporting-summary-flat.pdf

Life sciences study design

All studies must disclose on these points even when the disclosure is negative.

Sample size	We measured the entire isolate collection of the 1011 Saccharomyces cerevisiae genomes project.
Data exclusions	Gene copy numbers for natural isolates were downloaded from the 1002 Yeast Genome website, and the following loci were excluded: ribosomal DNA, Ty elements, RTM loci, ORFs located on the 2-micron plasmid, mitochondrial ORFs, and non-reference material. Furthermore, the table was filtered to only retain genes with non-zero and non-missing values for further analyses. Chromosome copy number status for all engineered disomic strains was confirmed by Torres et al. 2007, meaning all disomic strains used in our study were haploid with indicated “disomic” chromosomes duplicated. One exception was Disome 13: despite published mRNA expression values being available and proteomics data having been measured in our experiments, Disome 13 was excluded from all analyses due to having undergone whole-genome duplication when reaching our laboratory (private communication, J. Zhu). For 761 isolates, both proteomes (this study) and transcriptomes were available, and for 759 isolates, gene copy number information from Peter et al. 2018 was available. Data for gene copy number, mRNA expression, and protein abundances were matched by strain name and systematic ORF identifier for both the natural isolate collection and the disomic strain collection. Only genes for which values for gene copy number, transcript, and protein levels were available were used for analyses. We noticed that a number of strains in the natural isolate collection exhibited a mismatch between the median gene copy number per chromosome and the assigned aneuploidy, most likely attributable to segmental aneuploidies, shorter gene copy number variations, or algorithm-specific thresholds used for aneuploidy determination. We excluded all strains (n = 80) containing one or more of those “mismatched” chromosomes from our analysis.
Replication	The proteomic dataset for the small strain library (disomic lab-engineered strains) was acquired in triplicates, while the large dataset (> 1000 isolates) was acquired once. Here we determined reproducibility and technical precision through replicate injections of a reference sample (77 injections), similar to previous studies (e.g. Messner et al., Cell, 2023).
Randomization	The strains were arrayed in a randomized order.
Blinding	Blinding was not applicable since data acquisition was performed in an automated and randomized manner.

Reporting for specific materials, systems and methods

We require information from authors about some types of materials, experimental systems and methods used in many studies. Here, indicate whether each material, system or method listed is relevant to your study. If you are not sure if a list item applies to your research, read the appropriate section before selecting a response.

Materials & experimental systems

n/a	Involved in the study
☒	☐ Antibodies
☒	☐ Eukaryotic cell lines
☒	☐ Palaeontology and archaeology
☒	☐ Animals and other organisms
☒	☐ Clinical data
☒	☐ Dual use research of concern
☒	☐ Plants

Methods

n/a	Involved in the study
☒	☐ ChIP-seq
☒	☐ Flow cytometry
☒	☐ MRI-based neuroimaging
